# Highly-Controlled Soft-Templating Synthesis of Hollow ZIF-8 Nanospheres for Selective CO_2_ Separation and Storage

**DOI:** 10.1021/acsami.3c06502

**Published:** 2023-06-22

**Authors:** Fraz Saeed Butt, Allana Lewis, Riccardo Rea, Nurul A. Mazlan, Ting Chen, Norbert Radacsi, Enzo Mangano, Xianfeng Fan, Yaohao Yang, Shuiqing Yang, Yi Huang

**Affiliations:** † School of Engineering, Institute for Materials and Processes, The University of Edinburgh, Robert Stevenson Road, Edinburgh EH9 3FB, U.K.; ‡ Jiangsu Dingying New Materials Co., Ltd., Changzhou, Jiangsu 213031, China

**Keywords:** soft-templating, ultrasound-supported synthesis, hollow ZIF-8, CO_2_ selective separation and storage, adsorption/desorption cyclic performance

## Abstract

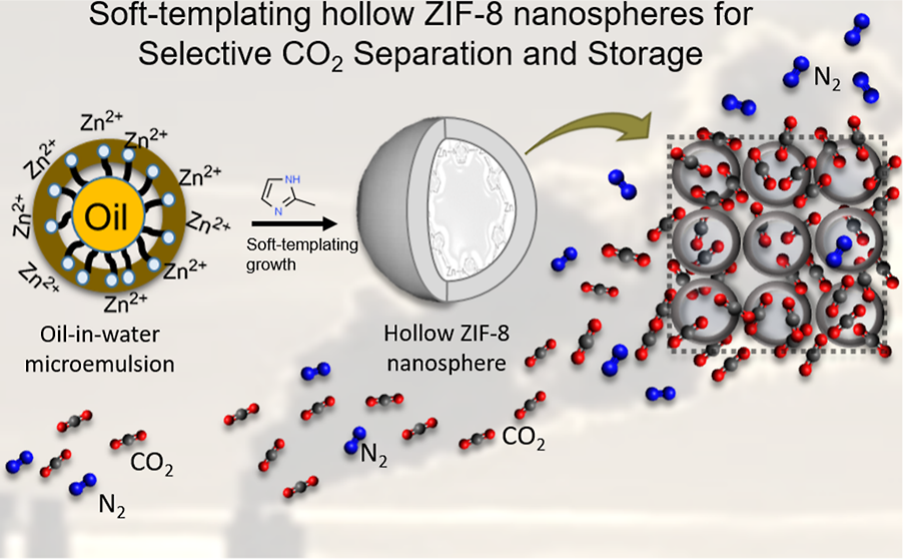

Global warming is an ever-rising environmental concern, and carbon dioxide (CO_2_) is among its major causes. Different technologies, including adsorption, cryogenic separation, and sequestration, have been developed for CO_2_ separation and storage/utilization. Among these, carbon capture using nano-adsorbents has the advantages of excellent CO_2_ separation and storage performance as well as superior heat- and mass-transfer characteristics due to their large surface area and pore volume. In this work, an environmentally friendly, facile, bottom-up synthesis of ZIF-8 hollow nanospheres (with reduced chemical consumption) was developed for selective CO_2_ separation and storage. During this soft-templating synthesis, a combined effect of ultra-sonication and low-temperature hydrothermal synthesis showed better control over an oil-in-water microemulsion formation and the subsequent growth of large-surface-area hollow ZIF-8 nanospheres having excellent particle size distribution. Systematic studies on the synthesis parameters were also performed to achieve fine-tuning of the ZIF-8 crystallinity, hollow structures, and sphere size. The optimized hollow ZIF-8 nanosphere sample having uniform size distribution exhibited remarkable CO_2_ adsorption capability (∼2.24 mmol g^–1^ at 0 °C and 1.75 bar), a CO_2_/N_2_ separation selectivity of 12.15, a good CO_2_ storage capacity (1.5–1.75 wt %), and an excellent cyclic adsorption/desorption performance (up to four CO_2_ adsorption/desorption cycles) at 25 °C. In addition, the samples showed exceptional structural stability with only ∼15% of overall weight loss up to 600 °C under a nitrogen environment. Therefore, the hollow ZIF-8 nanospheres as well as their highly controlled soft-templating synthesis method reported in this work are useful in the course of the development of nanomaterials with optimized properties for future CO_2_ capture technologies.

## 1 Introduction

The exponential increase in the atmospheric carbon dioxide (CO_2_) concentration due to the rise in the global population, which led to industrialization and excessive fossil fuel consumption, has raised significant environmental concerns regarding global warming.^1^ As per the global CO_2_ emission data reported in 2008, the 28,051 million metric tons (MMT) of CO_2_ emissions (recorded in the year 2005) are projected to increase to 42,325 MMT by 2030.^2^ Different techniques including adsorption,^3^ cryogenic separations,^4^ sequestrations,^5^ and absorption with suitable liquids^6^ have been adopted worldwide both at the commercial and industrial scale for effective CO_2_ separation, storage, and/or utilization. Among these techniques, CO_2_ separation and storage with nanoporous adsorbents have earned tremendous attention due to their facile synthesis procedure, large surface area, simplistic low-cost CO_2_ separation, and applicability over wide pressure and temperature ranges, owing to their structural flexibility.^7,8^

In recent decades, metal–organic frameworks (MOFs) have gained enormous attention.^9^ MOFs are famous for their unique characteristics including large surface area, ultrahigh porosity, tunability, and good structural stability and flexibility.^10^ The structural diversity of MOF results from the self-assembly of a huge selection of metal nodes/clusters and organic linkers.^11^ Therefore, MOFs are also referred to as porous-coordination-polymers, organic–inorganic hybrids, metal–organic polymers, and/or supramolecular structures.^12^ In addition, MOFs have shown benefits over conventional porous materials, such as zeolites, porous silica, and activated carbons, that suffered due to the lack of structural flexibility and tunability.^13,14^ For instance, their physicochemical properties can be systematically tailored for application-oriented MOF formations.^15,16^ Due to their rich structural diversity, MOFs have been widely introduced in many important industrial processes including separations,^17^ catalysis,^18^ drug delivery,^19^ biological^20^ and electrochemical sensing,^21,22^ and energy conversion.^23^

Zeolitic imidazolate frameworks (ZIFs), a requisite sub-category of MOFs, are formed through strong tetrahedral coordination between metal ions (Zn^2+^, Co^2+^) and imidazole linkers.^24^ Owing to this strong interaction, ZIFs exhibit higher structural stability than the majority of MOFs.^25^ Out of the entire ZIF family, ZIF-8 is the most studied nanostructured material and one of the few commercially available MOFs.^26^ ZIF-8 has a sodalite structure with a pore size of 3.4 Å, which is further connected to cavities with a size of 11 Å.^26^ The strong metal–ligand interaction in ZIF-8 results in good structural stability as compared to other ZIFs. Furthermore, ZIF-8 presents a large surface area, excellent pore size distribution, and good crystallinity.^26^ Owing to these unique characteristics, ZIF-8 has been extensively used for a wide range of applications in catalysis,^27^ gas separation,^28^ and storage.^29^ Additionally, ZIF-8 has been synthesized with various morphologies and nanostructures including zero-dimensional (0D) hollow structures,^30^ one-dimensional (1D) nanorods,^31^ two-dimensional (2D) nanosheets,^32^ and/or three-dimensional (3D) granular/spherical morphologies^33^ to better suit some specific applications. Among these morphologies, hollow ZIF nanostructures have the structural advantage of large interior storage cavities surrounded by a thin shell.^34^ These large-sized cavities provide excellent storage capability, while the presence of a thin outer shell allocates excellent sieving characteristics as well as minimal diffusional limitations. Therefore, these hollow nanostructures have been found promising for gas storage,^35^ catalysis,^18^ and drug delivery.^19^

To date, different strategies have been reported for the synthesis of hollow MOF structures. These strategies are classified based on template-free and templating (i.e., hard and soft template) growth.^33^ So far, MOFs have been synthesized using hard-templating routes due to easy control over the crystal growth and uniform particle size distribution (PSD).^36,37^ However, these synthesis routes often suffer economically due to their requirement for high synthesis temperatures and long synthesis times^34,38^ and also require post-synthesis processing or modifications for template removal.^33^ Unlike hard-templating growth, soft-templating synthetic routes do not require an energy-extensive process for template removal. This prevents possible shell structure damage of the hollow MOFs, which otherwise may result in the form of loss in crystal performance.^33^ Additionally, the soft-templating route has the advantages of simple fabrication procedures and efficient guest molecule encapsulation. However, soft-templating growth of 0D ZIF-8 nanospheres with highly controlled nanostructures has been rarely studied. Therefore, systematic studies on designing hollow ZIF-8 nanostructures via soft-templating synthesis routes are in urgent need.

Herein, we report a facile, one-pot, highly controlled soft-templating synthesis strategy for hollow ZIF-8 nanospheres with reduced synthesis time, energy consumption and overall chemical usage, ensuring the environmental friendliness of the synthesis procedure. Surfactant-stabilized oil-in-water (S-O/W) microemulsions (ME) were employed as soft templates for hollow ZIF-8 hydrothermal growth. It is worthwhile to mention that hydrothermal synthesis was chosen as it is preferred over solid-phase^39^ and gas-phase^40^ synthesis routes due to better control over final crystal size distribution, product purity, and operation simplicity.^40,41^ Furthermore, the effects of different synthesis parameters and conditions including the concentration of *n*-hexane and ligand, the mass of surfactant, order of chemical addition, and synthesis time and temperature on hollow ZIF-8 nanostructural properties, such as crystal size and morphology, relative crystallinity, surface area, thermal stability, and local chemical structures, were systematically studied. Moreover, the practical application of hollow ZIF-8 nanospheres in CO_2_ separation through adsorption experiments was demonstrated at two temperatures (0 and 25 °C) over a pressure range of 0–1.75 bar. In addition, the CO_2_/N_2_ adsorption selectivity was also evaluated and compared with traditional ZIF-8 crystals. Finally, the as-prepared ZIF-8 hollow spheres were studied for their CO_2_ adsorption capacity and cyclic adsorption/desorption performance (up to four CO_2_ adsorption/desorption cycles) at 25 °C. This work represents an important step forward toward a cost-effective, energy-efficient, and highly reproducible synthesis of hollow ZIF-8 spheres for selective CO_2_ separation and storage.

## 2 Experimental Section

### 2.1 Materials

2-methylimidazole (2-mim) (C_4_H_6_N_2_; 99%) and zinc nitrate hexahydrate [Zn(NO_3_)_2_·6H_2_O; 98%] were purchased from Acros Organics B.V.B.A. Chemicals Co. 1*H*,1*H*,2*H*,2*H*-Perfluorodecyltriethoxysilane (HFS) (C_16_H_19_F_17_O_3_Si; 97%), *n*-hexane (*n*-C_6_H_14_; 95%), herein referred to as “surfactant” and “oil/solvent” respectively, *c*-hexane (*c*-C_6_H_12_; 99.8%), and *n*-dodecane (*n*-C_12_H_26_; 99%) were provided by Alfa Aesar. Methanol (CH_3_OH; >99.8%), and ethanol (C_2_H_5_OH; >99.8%) was supplied by Fisher Scientific UK Ltd and used as received without further purification. De-ionized (DI) water was used in all the experiments.

### 2.2 Synthesis Procedures

**Figure 1:**
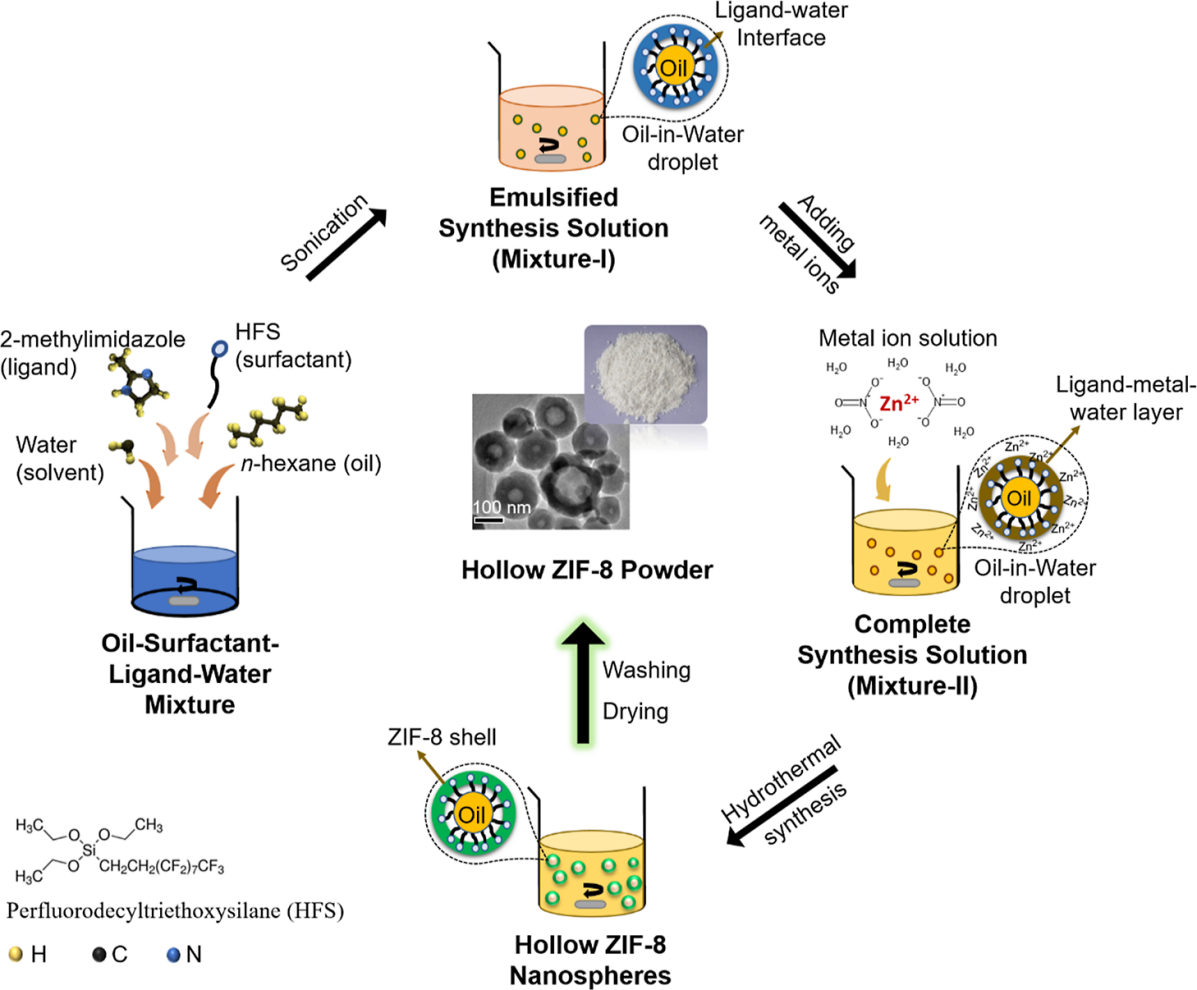
Soft-templating route for the synthesis of uniform hollow ZIF-8 nanospheres.

To analyze the effect of oil/solvent content, syntheses were carried out with 0, 0.5, 1, 2, 4, 8, and 12 mL of *n*-hexane, corresponding to 0, 1, 3, 5, 9, 17, and 23 v/v %, respectively. The surfactant HFS was deployed to stabilize the oil droplets within the water phase during ME formation. HFS was selected due to its superhydrophobic nature, giving greater stability to the overall oil-in-water ME (S-O/W).^42^ Additionally, the effect of surfactant within the system was investigated by varying its amount from 0 to 0.8 g. Both *n*-hexane and HFS, at the aforementioned concentrations, were added to a fixed 20 mL volume of ligand solution (13 g 2-mim in 400 mL DI water) to form mixture-I (Figure 1). Detailed information about the synthesis mixtures, prepared with different concentrations (v/v %) of *n*-hexane and surfactant wt %, is included in Tables S1–S3.

Subsequently, mixture-I was hard sonicated (2 L Digital Ultrasonic Cleaner Cavitek Professional, 100 W) for 2 h (as shown in Figure 1) before the addition of 20 mL metal solution (5.9 g of zinc nitrate hexahydrate in 400 mL DI water). The metal solution was added dropwise (to mixture-I) over 5 min to obtain mixture-II, which turned milky straightaway. The final synthesis mixture was maintained at 30 °C and 500 rpm for a range of synthesis times (0.5, 1, 1.5, 2, 6, 14, and 24 h). After the synthesis, the resultant mixture was centrifuged (Thermo Scientific IEC CL40) at 6000–6500 rpm and repeatedly washed using methanol. Finally, the samples were dried overnight using an air oven (Binder GmbH FD 115) at 60 °C.

### 2.3 Characterizations

The surface and cross-sectional structure of the prepared hollow ZIF-8 spheres was studied with a JEOL JSM-IT100 scanning electron microscope. The scanning electron microscopy (SEM) samples were prepared by adding a drop from the ZIF-8 powder-methanol mixture to the scanning electron microscope stub and drying for 2–3 h in an oven at 60 °C. Later on, the PSD of the hollow spheres was evaluated by using the SEM images and software ImageJ 2.3.0/1.53q (Fiji). The transmission electron microscopy (TEM) images were recorded using a JEOL transmission electron microscope-1400 to look into the morphology and hollow structure of the synthesized ZIF-8 spheres. The crystalline phase and crystallinity were tested with X-ray diffraction (XRD) analysis for the 2θ range of 5–40° with Cu Kα radiation using Bruker D2 Phaser. Fourier-transform infrared (FTIR) spectroscopy was performed using Nicolet iS10 in the range of 480–4000 cm^–1^ to determine the effect of synthesis composition on the local chemical structure of the as-synthesized hollow ZIF-8 spheres. Thermogravimetric analysis (TGA)/ differential scanning calorimetry (DSC) was performed (using TGA/DSC 3+ METTLER TOLEDO) for a temperature range of 25–800 °C at a heating rate of 10 °C min^–1^ under a nitrogen environment to evaluate the weight losses with temperature. The surface area and pore size distribution of the prepared hollow ZIF-8 spheres were determined with Autosorb iQ advanced micropore size and chemisorption analyzer (Quantachrome). The adsorption/desorption behavior was tested with N_2_ at 77 K for a pressure range of 1 × 10^–7^ bar to 1 bar. Before the N_2_ adsorption study, ZIF-8 powdered samples were degassed at 120 °C for 12 h to remove any moisture or guest molecules. The surface area was determined using the Brunauer–Emmett–Teller (BET) method. The micropore volume was determined by the t-plot, and the mesopore size distribution was calculated from the desorption branch using the Barrett–Joyner–Halenda method, assuming pore saturation. Visual representations of emulsion were collected using bright-field microscopy (Leica Epifluorescence Microscope). The Ossila contact analyzer (equipment and software) was used to evaluate the water contact angle (WCA) for studying the structural hydrophobicity.

#### 2.3.1 CO_2_ Adsorption Study

CO_2_ adsorption was performed using a Gemni-VII sorption analyzer at 0 °C and the constant pressure of 1 bar. Before the adsorption analysis, the samples were degassed at 120 °C for 12 h. The simplified form of the ideal adsorbed solution theory (IAST) equation was used for evaluating a binary mixture (CO_2_/N_2_) selectivity *S*_(1/2)_, where CO_2_ and N_2_ were designated as “1” and “2,” respectively, as shown in eq 1
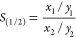
1where “*y*” and “*x*” are the molar fractions of the components in gas and the adsorbed phase, respectively.

#### 2.3.2 CO_2_ Adsorption Capacity and Cyclic Adsorption/Desorption Performance

Additionally, the hollow ZIF-8 samples’ CO_2_ adsorption capacity and recyclability were recorded by using TGA (TA instruments Trios V5). This was accomplished by loading the samples into the furnace at 25 °C under a nitrogen atmosphere (flow rate_Nitrogen_ = 25 mL min^–1^), and the change in mass was recorded at 0.1 data points/s. Then, the samples were heated linearly from 25 to 120 °C at 10 °C min^–1^ while maintaining the nitrogen atmosphere to exhaust. The sample was held isothermally at 120 °C for 4 h to reach equilibrium and cooled back to 25 °C in the nitrogen atmosphere. The gas flow rate was switched to carbon dioxide at 25 mL min^–1^ and held isothermally at 25 °C for 2 h while maintaining the CO_2_ atmosphere until the equilibrium is reached. This entire procedure was repeated four more times to test the adsorption capacity and the cyclic (CO_2_) adsorption/desorption behavior of the as-synthesized hollow ZIF-8 powder.

## 3 Results and Discussion

### 3.1 Pre-synthesis Ultrasonication

Initially, the effect of pre-synthesis ultrasonication on the growth of soft-templating hollow ZIF-8 spheres was studied by hard sonicating the ligand solution with the oil (*n*-hexane) phase. Herein, *n*-hexane was preferred due to its non-polar nature, long carbon chain (i.e., *n*-C_6_), and water immiscibility. This gives *n*-hexane the ability to form an oil-in-water emulsion to provide a soft template for ZIF-8 growth.^43^ More specifically, the sonication process enables high-energy ultrasonication-directed fragmentation of the oil layer into tiny oil droplets in water, thus creating a uniform oil-in-water ME.^44^ As a result, the sonication not only increased the surface area-to-volume ratio of the dispersed oil droplets but also promoted effective ligand–*n*-hexane interactions.^45,46^ Based on preliminary experiments, 2 h of pre-synthesis ultrasonication was found to be the minimum time required for a stable ME formation (Figure S1). Hereafter, the addition of metal ions initiates the ZIF-8 growth at the peripheral ligand/metal ions-water layer (Figure 1). Interestingly, no obvious crystallization was noticed in the absence of ultrasonication pre-treatment due to the absence of the ME growth template, as shown in Figure S1(I). The microscopic images for the oil-in-water (O/W) emulsion, prepared with 9 v/v % *n*-hexane and 2 h of pre-synthesis ultrasonication having an average droplet size of ∼3.40 ± 1.10 μm along with the droplet size distribution curve, are included in Figure S1(II,III), respectively.

### 3.2 Effect of Co-solvent Concentration

#### 3.2.1 Non-Surfactant-Stabilized Oil-in-Water Emulsion as the Growth Template

**Figure 2:**
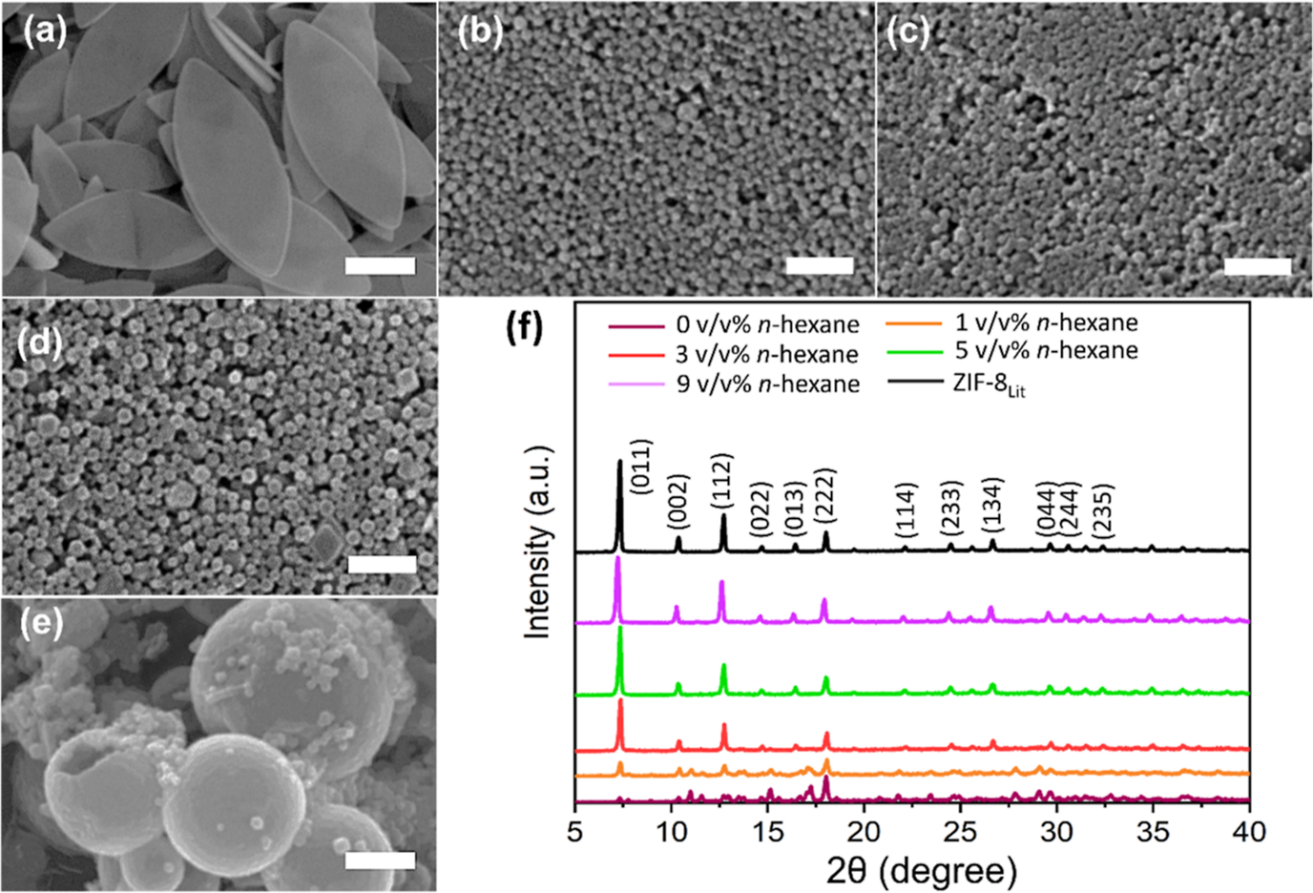
The SEM images showing the effect of variation in *n*-hexane’s volume fraction on ZIF-8 growth: (a) 0 v/v % (0 mL), (b) 1 v/v % (0.5 mL), (c) 3 v/v % (1 mL), (d) 5 v/v % (2 mL), and (e) 9 v/v % (4 mL) of *n*-hexane. (f) XRD pattern for 0, 1, 3, 5, and 9 v/v % of *n*-hexane in comparison to the XRD pattern for reference ZIF-8_Lit_ (all the scale bars are 2 μm).

Owing to the unique tunability and preferential formation of ZIF-8 crystals in the presence of various organic co-solvents,^38^ modifications were made to a previously reported ZIF-L recipe.^47^ As reported in our recent work,^48^ the introduction of an organic co-solvent to a preferential ZIF-L synthesis mixture favors the formation of a ZIF-8 phase with a significantly reduced ligand-to-metal (2-mim/Zn^2+^) ratio, for example, from 70 (required for traditional ZIF-8 synthesis) to 8 (the minimum ratio required for ZIF-L)^49^ at an optimized co-solvent concentration (v/v %). For this reason, a range of volume fractions of the soft-templating *n*-hexane equivalent to an overall 1, 3, 5, 9, 17, and 23 v/v % was studied to find an optimized oil concentration (v/v %) necessary for preferential ZIF-L → ZIF-8 phase change and the growth of ZIF-8 hollow spheres (for more information on sample preparation with various oil concentrations, see Table S1). The as-synthesized crystals were carefully analyzed with SEM and XRD to investigate the crystal morphology, crystallinity, and co-solvent-guided phase change (Figure 2).

Similar to our recent work, in the absence of any added organic co-solvent, the as-synthesized sample exhibited a 2D leaf-shaped morphology (Figure 2a), which is the typical ZIF-L morphology.^48^ Interestingly, at 1 v/v % (overall) concentration of *n*-hexane in mixture-I, a distinct change in crystal morphology to granular shape was observed, as shown in Figure 2b. This indicated that the presence of co-solvent played a crucial role in crystals’ morphological evolution.^48^ No significant change in crystal morphology was noticed with an increase in *n*-hexane concentration to 3 and 5 v/v %. All samples showed spherical crystals with slightly increasing sizes, from ∼220 nm (1 v/v %) to ∼260 nm (3 v/v %), and then to ∼350 nm (5 v/v %), as also shown in Figure 2b–d. The absence of any hollow structures in these samples indicated that lower oil contents favored the formation of solid ZIF nanocrystals. However, it was found that the further increase in the overall v/v % of *n*-hexane (≥9 v/v %) induced hollow structure formation. Figure 2e showed that a minimum of 9 v/v % of *n*-hexane was required to form a stable ME for initiating the growth of hollow structures with an average diameter of ∼2.50 ± 0.95 μm (Table S1) and a shell thickness of ∼0.25 ± 0.05 μm.

Although 9 v/v % of *n*-hexane helped to initiate a certain level of hollow structural formation, a broad PSD (∼0.50 to ∼5.5 μm) was observed, and the sample contained a majority of solid nanocrystals mixed with microsized hollow spherical particles. This is because of the ME instability and the possible re-fusion and accumulation of oil droplets to form larger ones during ZIF-8 synthesis. This re-fusion of oil droplets is evident from the SEM images, where the formation of large-sized ZIF-8 spheres (∼2.50 ± 0.95 μm) was noticed. In contrast, the rest of the reagents in the synthesis mixture still favored the formation of nano-ZIF-8s in the continuous aqueous phase. To promote hollow ZIF-8 growth, the concentration of oil (*n*-hexane) was further increased to 17 and 23 v/v % [Figure S2(I,II)]. The increase to 17 v/v % of *n*-hexane did not give a big difference as the sample showed a similar combination of solid nanocrystals and microsized hollow particles (Figure S2(I)). When the *n*-hexane volume ratio was increased to 23 v/v %, the sample with a more uniform size and narrow PSD was obtained (Table S1). However, this increase in the oil concentration still could not effectively avoid the formation of nano ZIF-8s due to the ME instability, as discussed above [Figure S2(II)]. Figure S2(III–V) shows the PSDs of the hollow spheres obtained for 9, 17, and 23 v/v % of *n*-hexane, respectively.

However, it is worthwhile to mention that the presence of *n*-hexane played a phase-directing role in ZIF-8 formation. This unique structure-directing phenomenon can be attributed to the ligand–(*n*-hexane) interaction, preventing the formation of hydrogen bonds between ZIF monolayers by pushing the ZIF monolayered structure away from each other.^38^ Such interactions facilitated the formation of new Zn–(2-mim)–Zn bonds, hence transforming the dimensionality from the 2D ZIF-L phase to the 3D ZIF-8 structure. The XRD results confirmed that only pure 2D ZIF-L was formed without *n*-hexane (Figure 2f). However, mixed ZIF-L/ZIF-8 phases were observed for the sample prepared with 1 v/v % of *n*-hexane. With a further increment in the *n*-hexane concentration, a pure ZIF-8 phase was formed in the synthesis with *n*-hexane concentrations ≥3 v/v % (i.e., ≥1 mL). The as-prepared samples displayed reflection along the planes (011), (002), (112), (022), (013), (222), (114), (233), (134), (044), (244), and (235), which is a characteristic ZIF-8 XRD pattern.^50^ The XRD results were also consistent with the reference ZIF-8 sample prepared through a previously reported aqueous phase synthesis,^51^ herein referenced as ZIF-8_Lit_. The recipe for the aqueous phase ZIF-8_Lit_ synthesis and the XRD pattern for the ZIF-8 samples synthesized with 17 and 23 v/v % of *n*-hexane are included in the Supporting Information [Method and Figure S2(VI)].

Overall, although 9 v/v % of *n*-hexane (ZIF-8_9 v/v%_) was found to be minimum for ZIF-8 hollow structural formation, the as-synthesized samples displayed a mixture of ZIF-8 nanocrystals and microsized hollow particles. Furthermore, a very low BET surface area of 685 m^2^ g^–1^ was obtained for ZIF-8_9 v/v%_ [Figure S2(VII) and Table 1] in comparison to the previously reported surface area of 1079 m^2^ g^–1^ obtained during an aqueous phase ZIF-8 synthesis.^52^ This is due to the lower crystallinity of the ZIF-8_9 v/v%_ sample as confirmed by the less intensified XRD diffraction peaks compared to those of the reference ZIF-8 sample. Longer synthesis time (>2 h) in the presence of *n*-hexane or further increment of the ligand-to-metal ratio in the synthesis solution is needed for the synthesis of well-crystallized ZIF-8 samples.^48^ However, as mentioned above, organic co-solvents (such as *n*-hexane) can promote ZIF-8 crystallization in an aqueous system with a significantly reduced ligand-to-metal (2-mim/Zn^2+^) ratio, on the other hand, reducing the chemical consumption in ZIF-8 synthesis.

**Table 1:** Structural Characteristics of the Hollow ZIF-8 Samples Prepared in This Work

sample	surfactant/oil ratio (S/O) (g mL^–1^)	morphology	BET surface area (m^2^ g^–1^)	pore diameter (nm)	total pore volume (cm^3^ g^–1^)
0 v/v % *n*-hexane		2D leaf-shaped	200	2.77	0.21
9 v/v % *n*-hexane		hollow + solid	685	1.08	0.38
ZIF-8-Out	0.075	hollow	1110	1.07	0.64
ZIF-8-Ins	0.075	hollow	1325	1.08	0.78

To further study the possibility of synthesizing uniform hollow ZIF-8 nanoparticles, surfactant (HFS)-stabilized emulsions (S-O/W) were then prepared and subsequently employed to enable soft-templating growth of hollow ZIF-8 nanoparticles with uniform sizes.

#### 3.2.2 Surfactant (HFS)-Stabilized Oil-in-Water Emulsion (S-O/W) as Hollow ZIF-8 Growth Template: Effect of the Amount of Oil Content

The effect of surfactant as an emulsion stabilizer was studied. As shown in Figure 1, a fixed amount (0.3 g) of surfactant (HFS) was introduced and added to mixture-I (containing 20 mL of ligand solution plus *n*-hexane) to promote surfactant-stabilized emulsion formation. To systematically analyze the effect of HFS surfactant on the soft-templating growth, the amount of surfactant was kept constant (0.3 g) while the relative v/v % of *n*-hexane was varied in a similar pattern as mentioned in Section 3.2.1. For example, the studied *n*-hexane concentrations include 3, 5, 9, 17, and 23 v/v %, respectively (for more information on the relative reagent concentrations of surfactant-supported synthesis solutions, please see Table S2 in the Supporting Information)

**Figure 3:**
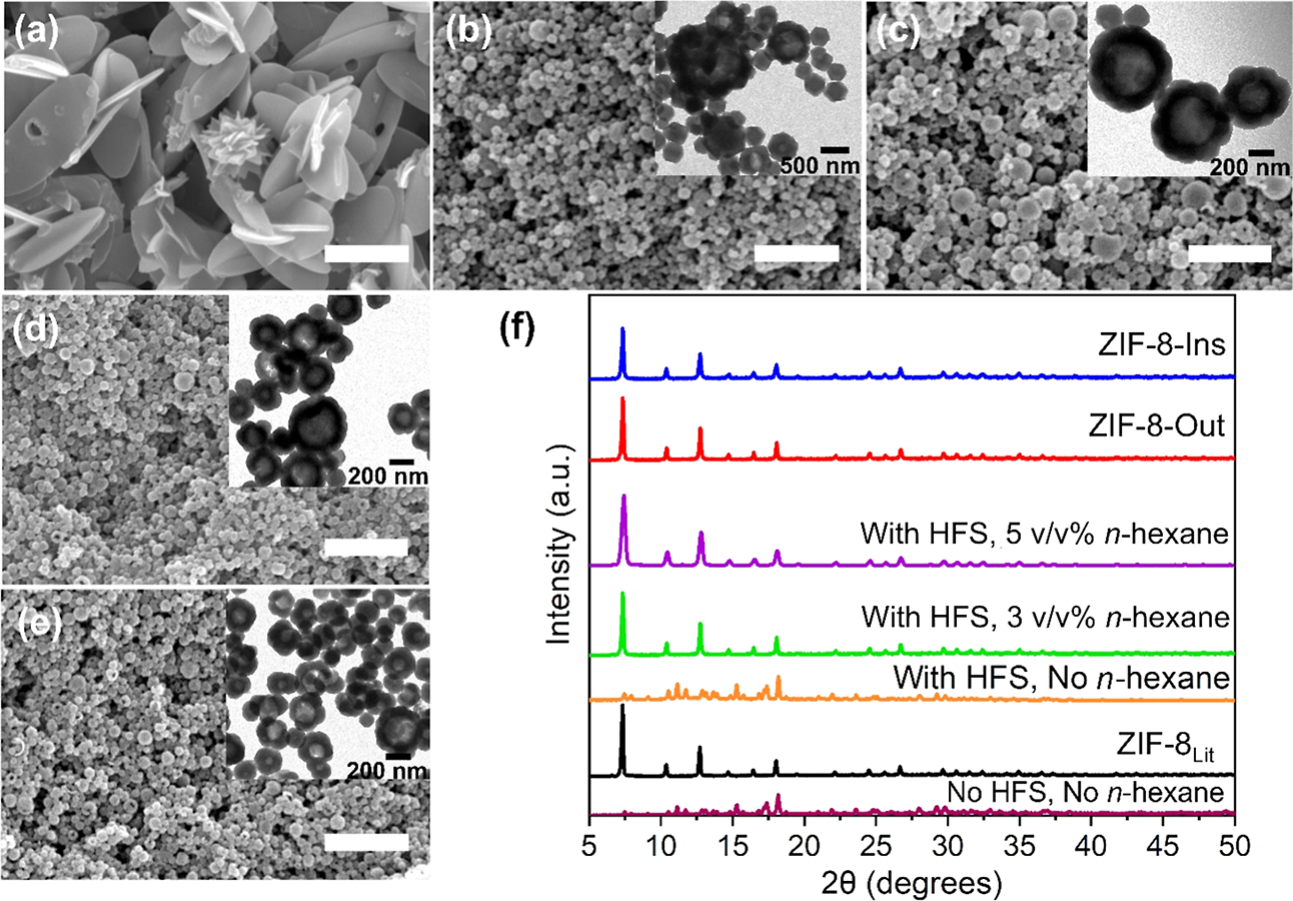
SEM images for (a) 0, (b) 3, (c) 5, (d) 9 (ZIF-8-Out), and (e) 9 v/v % (ZIF-8-Ins) mL concentration of *n*-hexane equivalent to the surfactant/oil (S/O) ratio of 0, 0.3, 0.15, 0.075, and 0.075 g mL^–1^, respectively, keeping the amount (0.3 g) of surfactant constant. The insets with figures (b–e) show the respective TEM images. (f) XRD pattern for the abovementioned samples (the scale bars are 5 μm unless specified in the image).

As seen in Figure 3a, the sample prepared with 0.3 g of HFS in the synthesis mixture (without *n*-hexane) exhibited a 2D leaf-shaped morphology, which is similar to previously reported ZIF-L crystals. The XRD results also confirmed the presence of a pure ZIF-L phase (Figure 3f), which ruled out the possibility of HFS as a ZIF-L → ZIF-8 phase directing agent. Interestingly, at low concentrations of *n*-hexane (3–5 v/v %) in the S-O/W emulsion, spherical particles with both solid and hollow structures appeared in the pure ZIF-8 phase (Figure 3b,c and insets). Compared to the samples prepared solely with *n*-hexane (see Section 3.2.1), the S-O/W emulsion showed the earlier onset of hollow particle formation even at low oil concentrations of 3–5 v/v % instead of 9 v/v %. More specifically, during surfactant-supported ME formation, the organic linker molecules were decorated around the ME peripheral interface, while the surfactant stabilized the emulsion with its hydrophobic end surrounding the oil droplet and the hydrophilic head interacting with the ligands and water molecules at the interface, stabilizing these oil droplets from fusing.^53,54^ This ME later served as an ideal template for the nucleation and crystallization of hollow ZIF-8 nanospheres.

Expectedly, the nanospheres prepared with 3–5 v/v % of *n*-hexane showed the presence of single-phase ZIF-8 (Figure 3f), further confirming the role of co-solvent in the ZIF-L → ZIF-8 phase direction (see Section 3.2.1). Surprisingly, when the oil was increased to 9 v/v %, under the same hydrothermal conditions, hollow ZIF-8 spheres (∼0.45 to ∼1 μm) were formed and dominant in the samples (Figure 3d,e and insets). The inset TEM images clearly showed a core–shell structure for the particle, with a large number of hollow ZIF-8 spheres falling within the nanoscale range (<500 nm). Compared to the sample prepared in O/W emulsion, the hollow particles showed a much smaller but more uniform size. This is due to the formation of uniform and small templating HFS–*n*-hexane micelles in the S-O/W emulsion (see Figure 1 for the possible structure of a single HFS–*n*-hexane micelle). Figure S3(I,II) shows the microscopic images of the S-O/W emulsion, prepared with 9 v/v % of *n*-hexane and 0.3 g of HFS in mixture-I, having an average droplet diameter of ∼0.95 ± 0.20 μm, and its related droplet size distribution, respectively. As discussed earlier, the HFS molecules with their hydrophobic end surrounding the oil droplet and the hydrophilic head interacting with the ligands and water molecules at the interface can, therefore, more effectively stabilize these oil droplets from fusing into bigger droplets. These HFS–*n*-hexane micelles thus served as an ideal template for the nucleation and crystallization of ZIF-8 on the peripheries forming a core/shell structure.

Overall, the ultrasonication-induced oil and surfactant interaction has proven effective in inducing hollow sphere formation. To further understand the effect of sonication on the oil–surfactant interactions, minor adjustments were made to the final mixing step—the addition of metal solution into mixture-I (Figure 1). Depending on whether the step was performed without or with sonication, the corresponding samples were named as ZIF-8-Out (Figure 3d) and ZIF-8-Ins (Figure 3e), respectively. In ZIF-8-Ins, unlike ZIF-8-Out, the metal solution was added dropwise to mixture-I during the last 10 min of pre-synthesis ultrasonication. After the addition of the metal solution, the mixture was transferred to a water bath for synthesis under the previously mentioned conditions.

Interestingly, the as-prepared ZIF-8-Ins displayed hollow sphere formations with excellent PSD and a least standard deviation value of only ∼0.08 (Table S2). The hollow spheres’ average diameter was further reduced to ∼0.40 μm (ZIF-8-Ins) compared to ∼0.75 μm (ZIF-8-Out), suggesting the influence of sonication on better forming ZIF-8 hollow sphere morphology. Furthermore, both ZIF-8-Out and ZIF-8-Ins showed an almost similar hollow sphere wall thickness equivalent to ∼0.08 ± 0.02 μm, suggesting the effect of surfactant (HFS) on the soft-templating oil-in-water spherical interface. Further discussion on the hollow ZIF-8 sphere wall thickness control is included in the Supporting Information. Figure 3d,e presents the SEM and TEM (insets) images for ZIF-8-Out and ZIF-8-Ins, respectively. More TEM images, verifying the hollow nature of both ZIF-8-Out and ZIF-8-Ins samples, are provided in the Supporting Information in Figure S3(III,IV), respectively. The SEM-based PSD curves for ZIF-8-Out and ZIF-8-Ins with a spherical diameter ranging between ∼0.45 to ∼1 μm (ZIF-8-Out) and ∼0.25 to ∼0.6 μm (ZIF-8-Ins) are shown in Figure S4(I,II), respectively.

Additionally, ZIF-8-Out and ZIF-8-Ins were analyzed with XRD to investigate the phase and relative crystallinity (Figure 3f). The obtained XRD patterns were in good agreement with those of the previously reported ZIF-8_Lit_.^52^ Furthermore, the relative crystallinity of the above-synthesized ZIF-8 samples was evaluated by calculating the area under the XRD curve. This estimation and comparison of the area under the curve is an indication of the formation of highly crystalline ZIF-8 nanocrystals.^55^ The area under the most prominent ZIF-8 XRD peak, corresponding to the (011) plane, was estimated for both ZIF-8-Out and ZIF-8-Ins samples. The ZIF-8-Out nanospheres exhibited a slightly higher value for the area under the curve (i.e., area_ZIF-8-Out_ = 355) in comparison to its counterpart ZIF-8-Ins (i.e., area_ZIF-8-Ins_ = 310) (Figure S4 (III,IV)), respectively, suggesting that ZIF-8-Ins displayed slightly lower crystallinity than ZIF-8-Out. This, as aforementioned, can be attributed to the high-intensity ultrasonic energy that not only promotes effective interaction between HFS, oil, ligands, and metal ions but also left some structural defects due to sonication-supported intensified rate of nucleation.^56,57^

**Figure 4:**
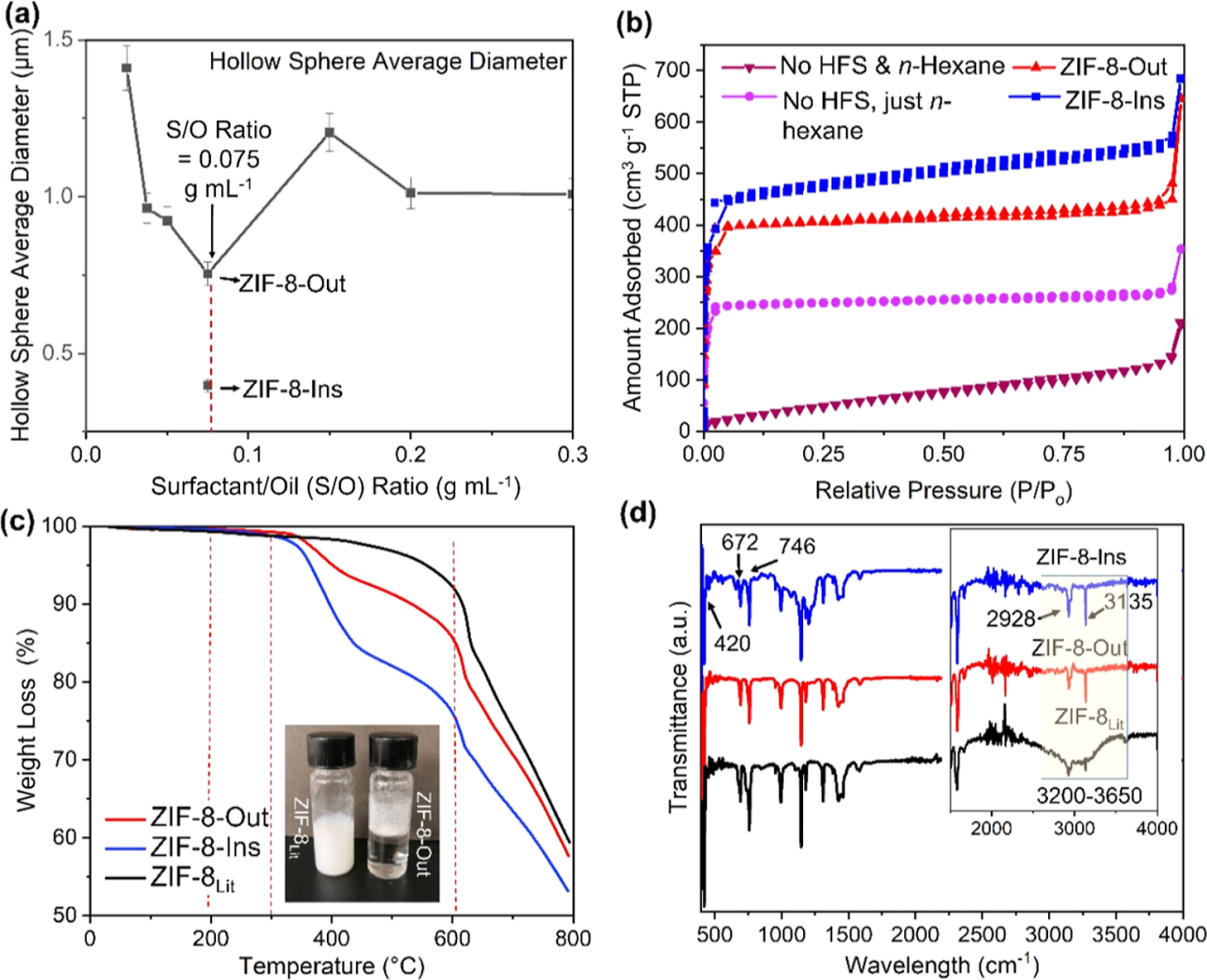
(a)Variation in the average diameter with a change in the surfactant/oil (S/O) ratio. (b) The BET adsorption isotherm for the samples ZIF-8-Out, ZIF-8-Ins, and the samples prepared with *n*-hexane and without surfactant and *n*-hexane. (c) TGA analysis for ZIF-8-Out and ZIF-8-Ins of reference. (d) FTIR spectra for the samples ZIF-8_Lit_, ZIF-8-Out, and ZIF-8-Ins; inset: enlarged region of the FTIR spectra between 1500 and 4000 cm^–1^ (inset: a photo of reference ZIF-8 sample and ZIF-8-Ins mixed with water).

Further increases in oil concentration to 13 v/v % (6 mL), 17 v/v % (8 mL), and 23 v/v % (12 mL) were also studied (Figure S5), and the sample properties (such as morphology and sphere diameter) are summarized in Table S2. Moreover, a summarized trend showing variation in the average diameter (evaluated from the SEM images using the software ImageJ) for different surfactant/oil (S/O) ratios was included in Figure 4a. The S/O ratio, which is defined as the ratio of surfactant mass (in grams) to oil volume (in mL), was developed to better understand the trend of hollow particle size along with the change of oil content in the system. Herein, the introduction of the S/O ratio allowed for careful manipulation of the relative percentages of the reagents in favor of hollow sphere synthesis. The optimization of the S/O ratio helped to estimate the favorable composition of the synthesis mixture for a highly controlled soft-templating growth of hollow ZIF-8 spheres (with excellent PSD) with reduced nano-ZIF-8 formations. For example, the S/O ratio is 0.05 (0.3 g/6 mL = 0.05 g mL^–1^) when 6 mL (13 v/v %) of *n*-hexane was used. Similarly, the S/O ratios for 8 mL (17 v/v %) and 12 mL (23 v/v %) of *n*-hexane are 0.0375 and 0.025 g mL^–1^, respectively. Generally, the higher the oil content, the smaller the S/O ratio in the synthesis solution. As summarized in Figure 4a, when increasing oil mass from 1 mL (3 v/v %) or decreasing the S/O ratio from 0.3 g mL^–1^, the sample showed decreased particle size. At the S/O ratio of 0.075 g mL^–1^ (or 4 mL of oil equivalent to 9 v/v % overall), the sample had the smallest particle size of ∼0.4 and ∼0.75 μm for samples prepared with (ZIF-8-Ins) or without (ZIF-8-Out) sonication applied to the final mixing step (metal solution addition to mixture-I). Further decreasing the S/O ratio to 0.025 g mL^–1^, or increasing oil volume to 12 mL (23 v/v %), led to an almost linear increase in hollow particle size. This was due to the variation in the *n*-hexane volume, which could affect the surfactant/oil (S/O) equilibrium. In the case of excess oil content, the amount of available surfactant was insufficient to counter all oil phases, and therefore, bigger oil droplets were formed for templating the growth of larger hollow ZIF-8. In this work, the best surfactant/oil ratio of 0.075 g mL^–1^ (ZIF-8-Out and ZIF-8-Ins) was proved to effectively promote the growth of uniform hollow ZIF-8 particles with the smallest sizes in the S-O/W emulsion. As discussed, the S/O ratio of 0.075 g mL^–1^ exhibited highly improved final hollow structures with minimal solid crystal formation. Any increase or decrease in the S/O ratio of S-O/W mixtures disturbed the synthesis equilibrium between the amount of surfactant and the volume of *n*-hexane added, hence resulting in significant variations in the average spherical diameters with increased standard deviation (Table S2).

Next, nitrogen sorption was employed to understand the physical structure of ZIF-8-Out and ZIF-8-Ins samples. They both showed significantly improved BET surface areas of 1110 and 1325 m^2^ g^–1^, respectively (Table 1). These surface areas were at least 1.5 times higher than that of the sample prepared without HFS (with just *n*-hexane) (i.e., 685 m^2^ g^–1^) and five times higher than that of the one prepared without both *n*-hexane and HFS (i.e., 200 m^2^ g^–1^) (Figure 4b and Table 1). More importantly, these samples even showed higher BET surface area than the ZIF-8 sample (1079 m^2^ g^–1^), which was prepared in aqueous solutions.^52^ Noticeably, the slightly higher surface area value for ZIF-8-Ins nanospheres can be attributed to the smaller spherical diameter (∼0.40 μm), improved PSD (st. dev._ZIF-8-Ins_ = ∼0.08), and possible sonication-induced structural defects. Möslein and co-workers reported that the ZIF-8 nanocrystals with missing metal and/or linkers in the structure showed enhanced structural reactivity and surface adsorption as the defective structure possessed an increased number of available vacant spaces.^58^ In this work, these structural defects might be generated due to the sonicated-induced enhanced nucleation rate for ZIF-8-Ins, which indeed showed a higher total pore volume (0.78 cm^3^ g^–1^) than ZIF-8-Out (0.64 cm^3^ g^–1^). Interestingly ZIF-8-Ins and ZIF-8-Out samples had almost doubled total pore volume in comparison to the sample prepared without *n*-hexane (0.38 cm^3^ g^–1^) (Table 1).

The thermal stability of the as-prepared ZIF-8-Ins and ZIF-8-Out was studied using TGA (Figure 4c). Both hollow samples showed excellent thermal stability, which is comparable to the reference ZIF-8 sample. Less than ∼1% weight loss was found for the temperature range of 0–200 °C under a nitrogen environment, indicating a small weight loss due to the removal of moisture, organics, and/or loosely adsorbed guest molecules (such as *n*-hexane and HFS).^59,60^ Before the onset of ZIF-8 structural collapse at ∼600 °C, both ZIF-8-Ins and ZIF-8-Out samples presented much more weight loss (∼15–25 wt %) than the reference ZIF-8 sample (∼10 wt %) due to the loss of oil/surfactant molecules, which were likely coordinated/trapped into ZIF-8 structures. More importantly, ZIF-8-Ins demonstrated ∼10% greater weight loss (<600 °C) compared with its non-sonicated counterpart (ZIF-8-Out). Similarly, the TGA-based DSC pattern showed a relatively higher weight loss for ZIF-8-Ins, for an equal amount of heat influx (W g^–1^) [Figure S5(IV)]. The higher weight loss for ZIF-8-Ins can be attributed to the decomposition of a greater amount of HFS molecules that were coordinated/trapped inside the hollow ZIF-8 particles during the synthesis. The secondary sonication during the mixing of metal solution and mixture-I containing HFS and *n*-hexane, therefore, was found to be very useful to effectively coordinate HFS in the S-O/W emulsion and promote uniform HFS–oil micelles for the soft-templating synthesis of ZIF-8-Ins. The presence of HFS, inside the hollow ZIF-8 particles, was further confirmed by the higher degree of WCA, owing to the superhydrophobic nature of HFS.^42^ The hollow ZIF-8 nanospheres showed superhydrophobicity with WCA as high as 150° in comparison to super hydrophilic ZIF-8_Lit_ (<10°) [Figure S5(V,VI)].

Finally, FTIR spectra of the ZIF-8-Ins and ZIF-8-Out were performed to study their chemical structures, which were then compared with the traditional ZIF-8 materials.^52^ As shown in Figure 4d, both hollow ZIF-8 samples exhibited characteristic ZIF-8 absorption bands. Aliphatic and aromatic C–H stretching peaks were observed at 3135 and 2928 cm^–1^, respectively, highlighting the presence of the imidazolate linker in the structure. sp^2^ C–H bending of the aromatic structure was witnessed at 672 and 746 cm^–1^. A strong Zn–N bond was found at 420 cm^–1^, confirming the coordination between the metal and ligand. Compared to the reference ZIF-8_Lit_ sample, the as-synthesized hollow spheres (ZIF-8-Out and ZIF-8-Ins) exhibited a low degree of −OH stretching bonds between 3200 and 3650 cm^–1^, which indicates that less moisture was adsorbed onto ZIF-8 structures. These results agreed with the above TGA studies, which showed less weight loss before 350 °C for ZIF-8-Out and ZIF-8-Ins. The surface property can be further verified by the fact that the as-synthesized hollow ZIF-8 samples were much more hydrophobic than reference ZIF-8, as shown by their WCAs [Figure S5(V,VI)]. Figure 4c (inset) showed that ZIF-8-Ins powder floated to the surface when mixing with water, while the reference ZIF-8 was readily mixed and dispersed in the water phase.

### 3.3 Effect of the Amount of HFS Surfactant

**Figure 5:**
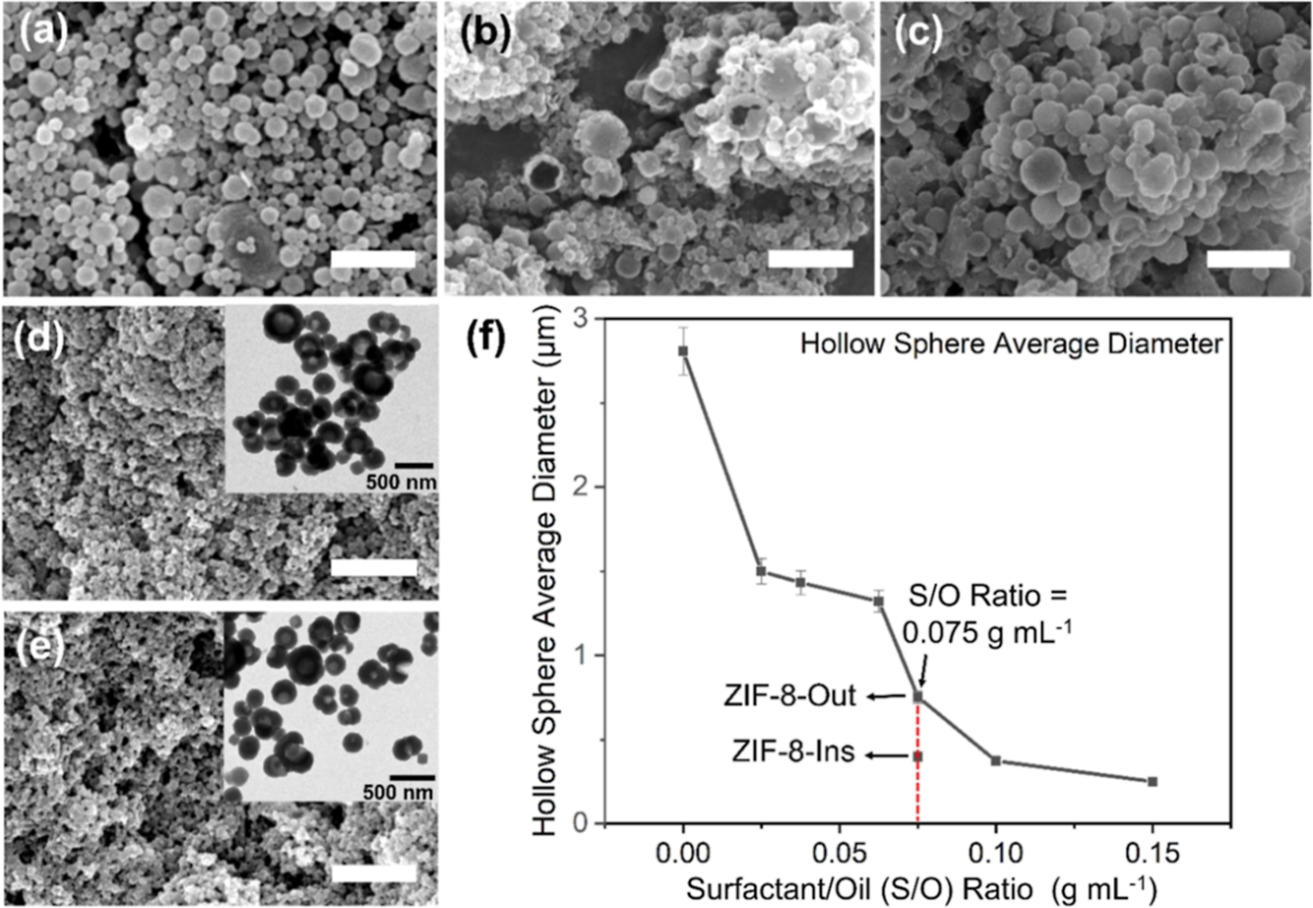
SEM images for (a) 0.10, (b) 0.15, (c) 0.25, (d) 0.40, and (e) 0.60 g of HFS equivalent to the surfactant/oil (S/O) ratio of 0.025, 0.037, 0.063, 0.10, and 0.15 g mL^–1^, respectively, keeping the volume of oil (*n*-hexane) constant at 4 mL (equivalent to 9 v/v %). The inset with figure (d,e) shows the respective TEM images. (f) Average diameter corresponding to each S/O ratio. (The scale bars are 5 μm unless specified in the image).

In Section 3.2.2 above, a fixed amount (0.3 g) of HFS surfactant was introduced to form a stable S-O/W emulsion with a varied amount of oil content. In this part, a fixed concentration of *n*-hexane (4 mL equivalent to 9 v/v %) was used, while the amount of the HFS surfactant was varied to study their effects on hollow ZIF-8 formation. More specifically, the quantity of HFS was varied for 0.10, 0.15, 0.25, 0.30, 0.40, and 0.60 g, which was equivalent to a S/O ratio of 0.025, 0.0375, 0.0625, 0.075, 0.10, and 0.15 g mL^–1^, respectively (Table S3). It was observed that only at the S/O ratio of 0.075 g mL^–1^ (0.3 g of HFS and 4 mL of *n*-hexane), the system could effectively produce the soft-templating growth of hollow ZIF-8 nanospheres having a narrow PSD, which was consistent with our previous study. The decrease in the S/O ratio below 0.075 g mL^–1^ (or the HFS mass loading <0.30 g) has led to the growth of comparatively large-sized hollow spheres with broad PSD due to the insufficient amount of surfactant, which affected the equilibrium between oil and surfactant and resulted in the re-aggregation of the oil droplets in the S-O/W emulsion. Figure 5a–c displays the SEM images for the samples prepared with 0.10, 0.15, and 0.25 g of HFS, respectively. An increase in ZIF-8 hollow sphere size was observed. Table S3 presents the PSD for the samples synthesized with variable amounts of HFS.

In contrast, the increase in the S/O ratio beyond 0.075 g mL^–1^ (or the HFS mass loading >0.30 g) resulted in a further reduction in particle size due to the increased amount of surfactant. ZIF-8 nanospheres prepared with a S/O ratio of 0.10 and 0.15 g mL^–1^ displayed a particle size equivalent to ∼0.37 ± 0.10 and ∼0.25 ± 0.10 μm, respectively (Figure 5d,e). The increased amount of surfactant and reduction in the average particle size promoted the formation of both solid and hollow ZIF-8 nanospheres, as confirmed by the TEM image (insets in Figure 5d,e). Moreover, a lower yield of hollow ZIF-8 particles was noticed. This indicated that the further increase in the amount of HFS (beyond 0.30 g) nullified the presence of *n*-hexane by reducing the interfacial tension between the oil and water phase, hence promoting the formation of a more solid ZIF-8 morphology.^61^ Hence, the S/O ratio of 0.075 g mL^–1^ proved to be an optimized ratio of the reagents, and any variation in the amount of surfactant or *n*-hexane disturbs the S-O/W equilibrium and promotes the formation of small-sized ZIF-8 crystallites with mixed morphology (Figure 5f).

### 3.4 Effect of Synthesis Conditions

After systematic studies on the effect of the system’s S/O ratio (or HFS and *n*-hexane content) on hollow ZIF-8 growth, synthesis conditions, such as synthesis time, temperature, and chemical addition order and concentration were also investigated to gain more understanding of the hollow ZIF-8 formation mechanism in S-O/W emulsion. Note, hereafter, the optimized S/O ratio of 0.075 g mL^–1^ was applied for the sample preparation under different synthesis conditions.

#### 3.4.1 Synthesis Time

As reported,^55^ synthesis time directly influences the MOF’s physicochemical properties. In this study, the synthesis time was varied in a wide range, for example, 0.5, 1, 1.5, 2, 6, 14, and 24 h for ZIF-8-Ins (*x* h) preparation with *x* denoting the synthesis time (0–24 h) (Table S4), and its influence on the characteristic properties of the as-synthesized samples was studied. The samples were studied mainly by SEM, XRD, TEM, and BET analyses. The most suitable synthesis time was selected based on the relative crystallinity, PSD, and surface area.

**Figure 6:**
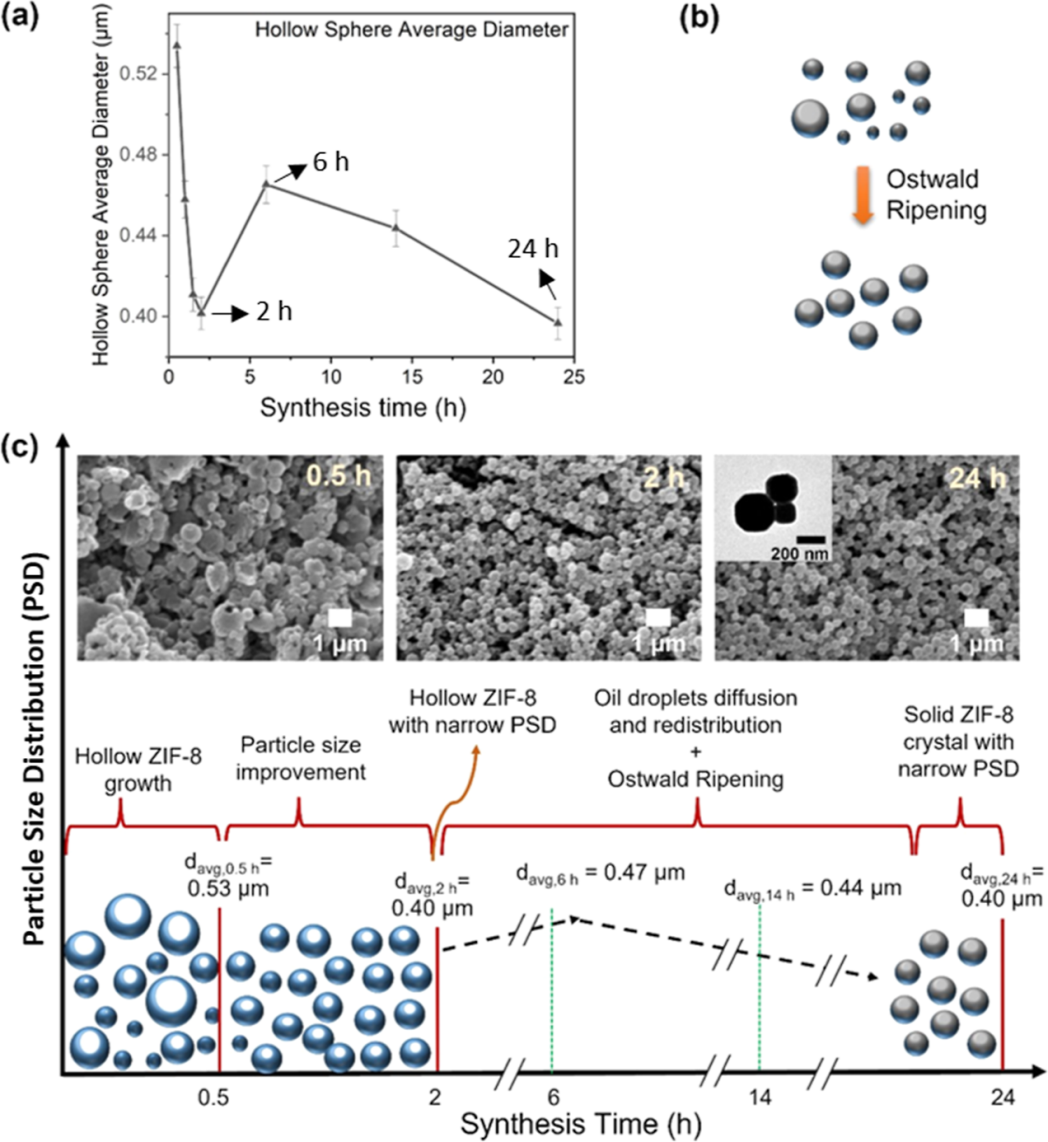
(a) SEM-based estimation of the average spherical diameter of ZIF-8 spheres for studied synthesis times, (b) Ostwald ripening, and (c) a summarized trend to explain the PSD based on synthesis time.

Based on the SEM images (Figure S6), the average hollow sphere diameter for each synthesis time was estimated. The sample synthesized for 0.5 h produced the largest-sized hollow spheres with an average diameter of ∼0.50 μm and exhibited the highest degree of standard deviation (∼0.30). Comparatively, after 2 h of synthesis, the sample showed the smallest hollow sphere diameter (∼0.40 μm) and the standard deviation (∼0.08). Between 0.5 and 2 h, a linearly decreasing trend for the average diameter and spheres’ standard deviation was noticed (Figure 6a and Table S4). This indicated that 2 h of synthesis time was needed for achieving a uniform hollow ZIF-8 formation. With a further increase in the synthesis time beyond 2 h, the prepared hollow spheres underwent an Ostwald ripening process until 24 h.^62^ The average hollow ZIF-8 nanosphere diameter after 6 and 14 h was ∼0.47 and ∼0.45 μm with a standard deviation of ∼0.12 and ∼0.10, respectively. During Ostwald ripening, the following two processes were expected to occur: (I) the oil droplets undergo oil diffusion and redistribution, where the small oil droplets coalesce to form bigger-sized droplets (Figure 6b); and (II) small hollow ZIF-8 nanospheres (≤∼0.3 μm) observed in sample ZIF-8-Ins (6–24 h) gradually dissolved for the growth of larger ZIF-8 crystals, as they were less thermodynamically stable than the larger crystals (Figures 6b and S6).^62^ As a result, the samples with longer synthesis time (>6 h) showed gradually improved PSD and more uniform particle size. For example, before 2 h of synthesis time, broad PSD was observed. However, at 2 h, ZIF-8-Ins (2 h) showed a much narrower PSD in the range of ∼0.25 to ∼0.6 μm (please see the Supporting Information Figure S7 for the PSD curve of each sample). After 2 h, due to the onset of the process (I), some larger hollow particles disassembled into smaller individual crystals, causing a temporary broadening of PSD, ∼0.15 to ∼0.80 μm [Figure S7(V,VI)]. Because of the ongoing process II (Ostwald ripening), small particles redissolved to provide supplemental nutrition for the growth of existing, more thermodynamically stable particles, forming more uniform crystals with similar PSD to that of hollow sample ZIF-8-Ins (2 h) [Figure S7(VII)].

In addition, Figure 6c graphically illustrated the particle size evolution for the synthesis up to 24 h, with some SEM images showing typical morphology and size at 0.5, 2, and 24 h. Interestingly, at 24 h of synthesis time, the as-obtained ZIF-8 nanospheres were mainly composed of solid ZIF-8 nanocrystals, suggesting the possible removal of the oil template during Ostwald ripening (between 2 and 24 h), as can be seen from the TEM image (inset Figure 6c). Moreover, the schematic illustration also showed the dynamic particle formation from large non-uniform core/shell morphology to uniform hollow spheres and then redissolved to form mostly solid ZIF-8 nanospheres with excellent PSD. Therefore, 2 h was found as an optimum value for the ZIF-8 hollow nanosphere synthesis through the soft-templating crystal formation mechanism in this work.

**Figure 7:**
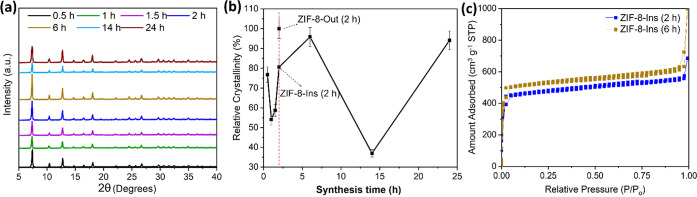
(a) Synthesis time-based XRD analysis, (b) XRD-based relative crystallinity analysis for 0, 0.5, 1, 1.5, 2, 6, 14, and 24 h, and (c) a BET adsorption/desorption isotherm for ZIF-8-Ins synthesized for 2 and 6 h.

Furthermore, the relative crystallinities for the samples were estimated and compared to find the optimal synthesis time for hollow particles. Figure 7a presents the XRD pattern observed for the studied synthesis times. The area under the (011) XRD data peaks from Figure 7a was measured, and the 6 h sample exhibited the maximum value for relative crystallinity with an area under the curve equal to ∼340. The XRD-based area under the curve calculated for each synthesis time is included in Table S5 in the Supporting Information. After 6 h, a clear decrease in relative crystallinity was recorded for 14 h with an area under the curve equal to ∼130 and then increased back to ∼335 for 24 h. This can be attributed to the particle size and morphological evolution (as discussed above).

Overall, the relative crystallinity followed a zig-zag trend of increasing and decreasing relative crystallinities, as was observed for PSD (Figure 7b). Further, the large BET surface area of 1470.30 m^2^ g^–1^ and enhanced total pore volume (0.95 cm^3^ g^–1^) for the 6 h sample can be attributed to the well-developed highly crystalline structure (Figure 7c), supporting the large XRD-based area under the (011) peak. However, the 6 h sample suffered from inconsistent PSD with a standard deviation of ∼0.12 (Tables S4 and S5).

Interestingly, the 2 and 24 h of synthesis times presented an area under the curve equivalent to ∼310 and ∼335, respectively. Due to such a small difference in the relative crystallinity, the 2 h of synthesis time can be regarded as an optimum synthesis time for hollow ZIF-8 nanosphere formation. A detailed relative crystallinity analysis based on the area under the XRD curve is provided in Figures S8 and S9. As noticed, the nanospheres obtained after two 2 h of synthesis have the advantages of excellent crystallinity (similar to 24 h) and a large surface area (similar to 6 h). As a result, the 2 h of synthesis time was finalized as the optimum time required for hollow ZIF-8 formation with uniform particle size and was considered for subsequent studies.

#### 3.4.2 Synthesis Temperature

The synthesis temperature has a significant effect on the crystal size and PSD of the synthesized samples. The elevated temperatures help to promote fast nucleation during the earlier stage of synthesis.^24^ Here, for example, the formation of hollow spheres appear to be significantly enhanced, with more frequent hollow spheres observed at relatively higher synthesis temperature. More specifically, for S/O mixtures (prepared with 9 v/v % of *n*-hexane without the addition of surfactant HFS), the sample demonstrated an increased number of hollow sphere formations with a relatively smaller crystal size, reducing from ∼2.50 ± 0.95 to ∼1.80 ± 0.30 μm by simply increasing the temperature from 30 to 50 °C (Table S6). Notably, despite the smaller crystal size, the PSD showed a lower deviation [Figure S10(I)]. This can be attributed to the faster rate of nucleation around the oil droplets at increased synthesis temperature,^63^ preventing the re-fusion of emulsified drops of oil in O/W mixtures. Although the quality of the hollow sphere crop was upgraded, the formation of nanosized solid ZIF-8s was still unavoidable. This was due to the lower stability of the prepared ME (as discussed above), indicating a requisition for emulsion stabilization by adding HFS as a stabilizer.

The effect of synthesis temperature was also noticed for the S-O/W mixture at a S/O ratio of 0.075 g mL^–1^ [Figure S10(II)], which proved to be the best S/O ratio for hollow ZIF-8 formation (as discussed above). It was observed that the average diameter of the prepared hollow spheres was lower (i.e., average diameter_ZIF-8-Out, 50°C_ = ∼0.65 ± 0.10 μm) than ZIF-8-Out (i.e., average diameter_ZIF-8-Out, 30°C_ = ∼0.75 ± 0.14 μm). However, it did not seem to have any significant influence on the sphere size standard deviation (Table S6). This relatively lower spherical diameter for ZIF-8-Out at 50 °C can be attributed to the enhanced rate of nucleation, resulting in the formation of small-sized crystallites due to the lack of material aggregation. On the other hand, the sample prepared inside the sonication bath still exhibited better PSD with a smaller average diameter (i.e., average diameter_ZIF-8-Ins, 30°C_ = ∼0.40 μm). The average diameter of the as-synthesized hollow nanospheres followed the order of ZIF-8-Out @ 30 °C > ZIF-8-Out @ 50 °C > ZIF-8-Ins @ 30 °C, which further highlighted the significance of the ultrasonication bath in controlling the overall PSD. Second, the samples, ZIF-8-Out @ 50 °C and ZIF-8-Ins @ 30 °C shared an almost similar TGA pattern [Figure S10(III)]. Therefore, it was noticed that the increment in the synthesis temperature does not have any significant influence on the structural properties of the prepared samples. As a result, the synthesis temperature of 30 °C proved to be sufficient for the ZIF-8 synthesis.

#### 3.4.3 Order of Reagent Addition and Ligand Concentration

In the last study, the effect of the ligand’s order of addition was investigated by swapping the ligand in the S-O/W mixture with the metal solution. The mixture containing oil, surfactant, and metal ions was sonicated for 2 h before the dropwise addition of the ligand solution. However, the SEM images in Figure S11(I) in the Supporting Information present the formation of a leaf-shaped morphology. Another attempt was made to stabilize the ME by doubling the amount of surfactant. Yet, it did not prove effective in hollow sphere synthesis [Figure S11(II)]. Instead, solid spherical morphology was observed. This indicated that the metal solution was not able to bond with oil and the surfactant molecules as easily as a ligand. This was because, unlike metal, ligand molecules could easily bond with the oil and surfactant molecules through CH−π and π–π interactions,^64,65^ leading to the deposition of the ligand molecules around the periphery of the ME, which resulted in hollow ZIF-8 morphologies. It is interesting to note that the order of reagent addition not only provided extra stability to the ME but also promoted the ZIF-8 growth with a ZIF-L recipe.

Finally, the increased (doubled) ligand concentration (in mixture-I) and its influence on hollow ZIF-8 synthesis were studied, while the rest of the synthesis parameters were kept constant. Expectedly, the as-synthesized sample showed a very uniform spherical morphology with a small crystal size [Figure S11(III)]. This is because the increased ligand concentration favors the growth of ZIF-8 crystal in the solution.^51^Figure S11(IV) shows the TEM image for elaborating the formation of solid ZIF-8 crystallites, with an exception of a few hollow sphere formations. The optimized hollow ZIF-8 synthesis conditions were also studied for other co-solvents having different carbon chain lengths (ethanol and *n*-dodecane) and structural configurations (*c*-hexane), and the relevant discussion is included with Figure S12.

### 3.5 Adsorption Isotherms: CO_2_ Selectivity and Cyclic Performance

#### 3.5.1 CO_2_ Adsorption and Selectivity

**Figure 8:**
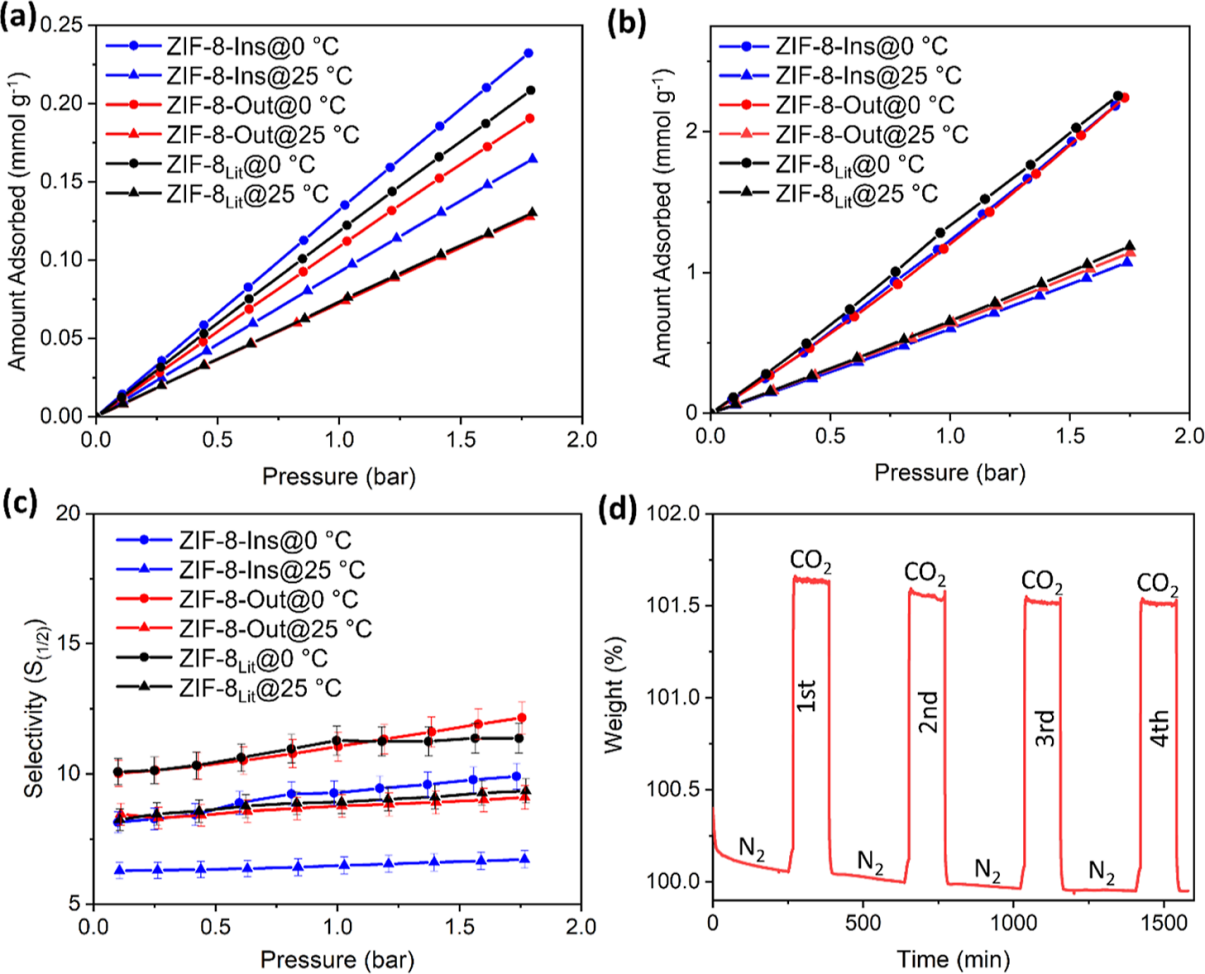
(a) N_2_, (b) CO_2_, and (c) CO_2_/N_2_ adsorption selectivity evaluated using the IAST model, respectively, for ZIF-8-Ins, ZIF-8-Out, and ZIF-8_Lit_ at 0 °C (circles) and 25 °C (triangles); (d) the CO_2_ recyclability data obtained at 25 °C, showing both CO_2_ adsorption capacity as well as cyclic adsorption/desorption performance up to four cycles.

The N_2_ and CO_2_ adsorption isotherms were recorded for the hollow ZIF-8 nanospheres synthesized with a S/O ratio of 0.075 g mL^–1^, including ZIF-8-Ins and ZIF-8-Out, to study their gas adsorption characteristics. The adsorption behavior was compared with the ZIF-8_Lit_ sample.^52^ Initially, the samples were degassed at 120 °C for 12 h and the N_2_ and CO_2_ isotherms were recorded at 0 and 25 °C while the gas pressure was varied between 0 and 1.75 bar. Span and Wagner^66^ and Kunz and Wagner^67^ equations of state were applied to evaluate the N_2_ adsorption isotherms, whereas, Span and Wagner^68^ equations were used for estimating CO_2_ adsorption. For a better comparison, the adsorption isotherms for both N_2_ and CO_2_ obtained at different temperatures (i.e., 0 and 25 °C) are provided in Figure 8a,b, respectively. The temperature of 0 °C was selected for evaluating the maximum (CO_2_) adsorption capacities of the powdered adsorbents for a systematic comparison with the previously reported data. Moreover, the adsorption temperature of 25 °C emulates the normal room temperature adsorption. Expectedly, the powdered samples presented greater N_2_ and CO_2_ adsorption at 0 °C than at 25 °C. This suggested that the adsorption of both N_2_ and CO_2_ is necessarily a physical phenomenon.

Figure 8a presented a higher N_2_ sorption capacity (∼0.23 mmol g^–1^ at 0 °C and ∼0.17 mmol g^–1^ at 25 °C) for ZIF-8-I than the other samples analyzed (i.e., ZIF-8-Out and ZIF-8_Lit_). This can be attributed to the large surface area and better PSD value for ZIF-8-Ins. Comparatively, ZIF-8_Lit_ demonstrated N_2_ sorption equivalent to ∼0.21 mmol g^–1^ at 0 °C and ∼0.13 mmol g^–1^ at 25 °C, whereas ∼0.19 mmol g^–1^ at 0 °C and ∼0.13 mmol g^–1^ at 25 °C were recorded for ZIF-8-Out. Expectedly, the studied samples presented a relatively higher affinity for CO_2_ than N_2_ (Figure 8b), which in turn helped in achieving larger CO_2_/N_2_ selectivity, for highly selective CO_2_ separation. The CO_2_ sorption capacity was within the range of ∼2.12–2.24 mmol g^–1^ at 0 °C, which decreased to about ∼1.02–1.14 mmol g^–1^ at 25 °C. This CO_2_ adsorption behavior is in good proximity to the results reported previously in the literature (Table S7). This further confirms that the samples ZIF-8-Ins and ZIF-8-Out can be effectively used for highly efficient CO_2_ separation. Moreover, the requirement for a lower synthesis temperature and time, in addition to the low ligand and solvent consumption for desired ZIF-8 formations, improves the overall process economics and environmental friendliness (Table S8).

The IAST model, developed by Myers and Praunitz,^69^ was used to evaluate the CO_2_/N_2_ selectivity. Equation 1(8) was applied to estimate the CO_2_/N_2_ selectivity values at 0 and 25 °C for ZIF-8-Ins, ZIF-8-Out, and ZIF-8_Lit_. The estimated adsorption selectivities are provided in Figure 8c. All the analyzed samples displayed a linear trend for selectivity, with ∼12.15 recorded as the highest selectivity value for ZIF-8-Out at 0 °C. As expected, the selectivity values were higher at lower temperatures due to enhanced adsorption. Additionally, the selectivity values were comparable to the highly crystalline ZIF-8_Lit_. Surprisingly, the sample ZIF-8-Ins exhibited slightly lower selectivity values in comparison to ZIF-8-Out and ZIF-8_Lit_. This is because the as-synthesized ZIF-8-Ins displayed good adsorption capacities for both N_2_ and CO_2_, resulting in reduced CO_2_/N_2_ selectivity. Overall, the higher CO_2_/N_2_ selectivity for the above-tested samples is the result of favorable CO_2_-to-ZIF-8 framework interaction than N_2_.^70^ In the following section, the CO_2_ adsorption capacity and the cyclic adsorption/desorption performance will be evaluated at 25 °C.

#### 3.5.2 CO_2_ Adsorption Capacity and Cyclic Adsorption/Desorption Performance

Furthermore, replicating the room temperature storage conditions, the hollow ZIF-8 nanostructures were tested for their CO_2_ storage capacity at 25 °C and cyclic adsorption/desorption performance using TGA. The samples were initially degassed by linear heating of the samples from 25 to 120 °C at a heating rate of 10 °C min^–1^ under a nitrogen flow rate of 25 mL min^–1^. The sample was kept isothermally under nitrogen flow for 4 h at 120 °C before switching to CO_2_ (flow rate_Carbon dioxide_ = 25 mL min^–1^). A ∼1.5–1.75 wt % increase in the overall weight of the analyzed sample was recorded under a CO_2_ flow rate of 25 mL min^–1^ at 25 °C for 2 h until equilibrium was reached. Interestingly, the sample presented excellent cyclic performance, up to four cycles of CO_2_ adsorption/desorption performed in this work, with absolutely no loss in the gas adsorption performance between each measurement (as shown in Figure 8d).

## 4 Conclusions

In conclusion, we report a facile, one-pot, bottom-up synthesis for hollow ZIF-8 nanospheres through a soft-template growth route. The ultrasonication-assisted hydrothermal synthesis has been adopted, and the effect of variations in synthesis conditions and parameters was investigated. Two hours of strong sonication and a S/O ratio of 0.075 g mL^–1^ followed by 2 h of synthesis at 30 °C were found to be the optimal conditions for hollow ZIF-8 nanosphere synthesis. The as-prepared samples exhibited a large surface area (∼1325 m^2^ g^–1^) and pore volume (∼0.78 cm^3^ g^–1^). Additionally, the nanospheres exhibited an excellent PSD, good crystallinity, and remarkable thermal stability. Interestingly, the hollow sphere formation required a much shorter synthesis time and lower temperature in comparison to the previously reported literature on hollow ZIF-8 synthesis (Table S8). The synthesized samples were also tested for CO_2_ separation and storage. The CO_2_ adsorption capacity was recorded as high as ∼2.24 mmol g^–1^ of CO_2_ at 0 °C and 1.75 bar pressure with a CO_2_/N_2_ selectivity of ∼12.15, which were in good agreement with the results reported previously in the literature (Table S7). The material exhibited excellent cyclic adsorption/desorption performance with no obvious reduction in CO_2_ adsorption capacity and a good CO_2_ storage capacity (∼1.5–1.75 wt %). In addition, this work provided a rationale and detailed understanding of the effect of the growth parameters on hollow ZIF-8 formations with reduced chemical usage, making it an ecofriendly and economical synthesis. Therefore, the hollow ZIF-8 nanospheres, as well as their highly-controlled soft-template synthesis method reported in this work, are useful in the course of the development of nanostructured materials with optimized properties for future CO_2_ capture technologies.

## Supporting Information Available

Synthesis solutions, hollow ZIF-8 sphere sizes and comparison with previous literature, microscopic images of the O/W and S-O/W emulsions, SEM and TEM images, SEM-based sphere size distribution curves, XRD spectra, XRD-based area under the peak comparison curves, TGA, DSC, water contact angle (WCA), and N_2_ adsorption/desorption isotherms (PDF)
