# Supplementary material for: Highly-Controlled Soft-Templating Synthesis of Hollow ZIF-8 Nanospheres for Selective CO2 Separation and Storage
Source: ACS Appl Mater Interfaces. 2023 Jun 22;15(26):31740–54. doi: 10.1021/acsami.3c06502 (PMC10326808; doi:10.1021/acsami.3c06502)
Supplement: Supplementary file 1 — am3c06502_si_001.pdf [file am3c06502_si_001.pdf]

Supporting Information

Highly-Controlled Soft-Templating Synthesis of Hollow ZIF-8 Nanospheres for Selective CO_2 Separation and Storage

*Fraz Saeed Butt,[1] Allana Lewis,[1] Riccardo Rea,[1] Nurul A. Mazlan,[1] Ting Chen,[1] Norbert Radacsi,[1] Enzo Mangano,[1] Xianfeng Fan,[1] Yaohao Yang,[2] Shuiqing Yang,[2] Yi Huang[1,]**

[1]School of Engineering, Institute for Materials and Processes, The University of Edinburgh, Robert Stevenson Road, Edinburgh, EH9 3FB, United Kingdom.

[2]Jiangsu Dingying New Materials Co., Ltd., Changzhou, Jiangsu, 213031, China.

Corresponding author: Dr Yi Huang, E-mail: Yi. Huang@ed.ac.uk

Methods

Fabrication recipe for ZIF-L

The ZIF-L nanocrystals were synthesized using a recipe reported in the previous literature.[1] Briefly, the 'metal' and 'ligand' solutions were prepared separately by adding 0.59 g of zinc nitrate hexahydrate ($Zn(NO_3)_2.6H_2O$) and 1.3 g of 2-methylimidazole (2-min) in 40 mL of DI water each, respectively. Later, the 'metal solution' was added drop-wise to the ligand solution and the fabrication mixture was left for 2 h at room temperature (22 ± 2°C) and 500 rpm.

Fabrication recipe for ZIF-8_{Lit}

The recipe for an aqueous phase synthesis of ZIF-8_{Lit} was taken from previously reported literature.[2] The 'metal' and 'ligand' solutions were prepared separately by adding 1.17 g of zinc nitrate hexahydrate ($Zn(NO_3)_2.6H_2O$) in 8 g DI water and 22.7 g of 2-methylimidazole (2-mim) in 80 g DI water, respectively. The 'metal' solution was added to the 'ligand' solution instantly and the mixture was left at room temperature (22 ± 2 °C) and 500 rpm. The synthesis recipe was slightly modified and the synthesis time was increased to 2 h. This allowed for a better comparison with the current fabrications.

The synthesized powders were separated by centrifugation and washed several times using methanol. Finally, the obtained powder was dried overnight using an air oven at 60 °C.

Hollow ZIF-8 Sphere Wall Thickness Control

In the current synthesis of soft-templating hollow ZIF-8, the addition of surfactant (HFS) significantly reduced the hollow sphere shell thickness. For instance, the addition of 0.3 g of HFS decreased the shell thickness from ~0.25 ± 0.05 µm (for just 9 v/v% of *n*-hexane, without any surfactant) to ~0.08 ± 0.02 µm for the as-obtained ZIF-8 hollow spheres (prepared with 9 v/v% of *n*-hexane and 0.3 g of surfactant). This reduction in the shell wall thickness highlights the role of surfactants in better-forming stable and uniform oil-in-water emulsion templates. Similar results were reported by Salihovic et al. where the hollow sphere shell thickness of carbon spherogels was dependent upon the amount of hard templating polystyrene (PS), where an increased template concentration resulted in a reduced hollow sphere shell thickness.[3] It is also worth noting that the addition of HFS not only helps the formation of significantly more oil-in-water nano-templates but also improves their stability during the hollow structure formation. This is crucially important to control the shell thickness and contributes to the excellent uniformity of the hollow particle size eventually. In contrast, in the syntheses without HFS, a broad particle size distribution as well as a wide range of shell thickness were observed. This is because the oil/water emulsion was formed without HFS templating at the oil/water interface, thus less stability of the emulsion was observed, resulting in less stable and non-uniform oil/water templates for hollow ZIF-8 growth which showed uncontrolled particle size and shell thickness.

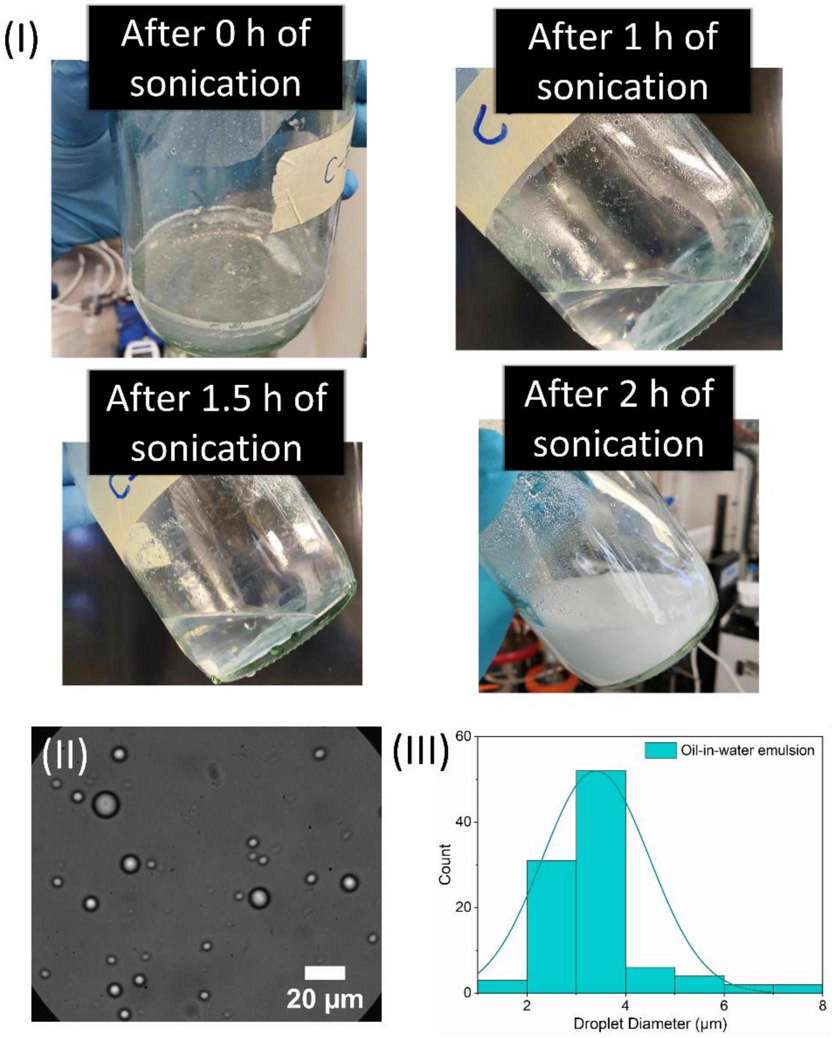

Figure S1. (I) Synthesis mixtures obtained after different pre-synthesis sonication durations (for oil-in-water emulsion formation). **(II)** The microscopic image for oil-in-water emulsion prepared with 9 v/v% (4 mL) of *n*-hexane in Mixture-I. **(III)** Droplet size distribution for oil-in-water emulsion.

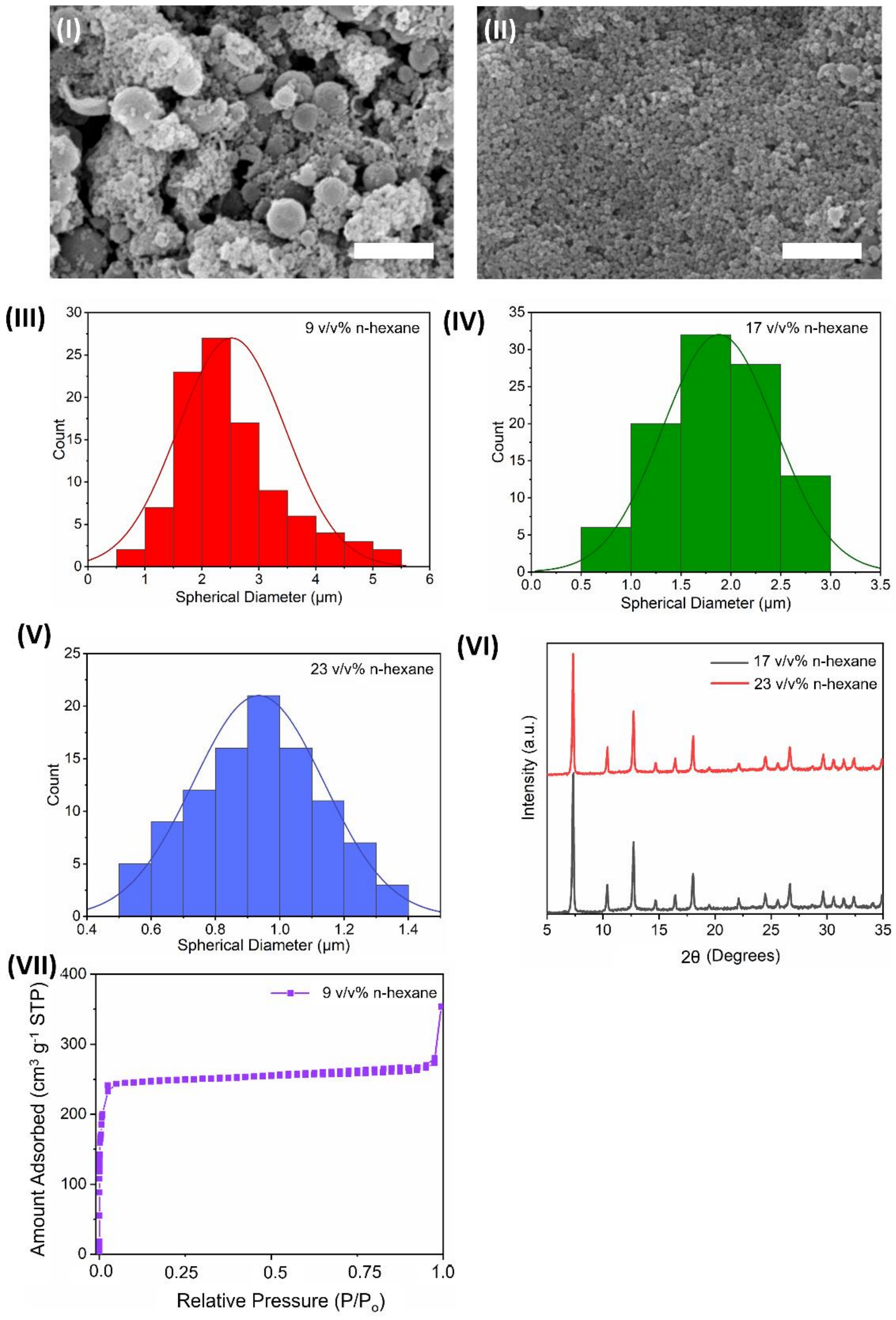

Figure S2. SEM images for samples prepared with **(I)** 17 v/v% (8 mL) and **(II)** 23 v/v% (12 mL) *n*-hexane, respectively. Particle size distribution for **(III)** 9 v/v%, **(IV)** 17 v/v%, and **(V)** 23 v/v% of *n*-hexane. **(VI)** XRD pattern and **(VII)** BET adsorption isotherm. (Scale bars are 5 μm)

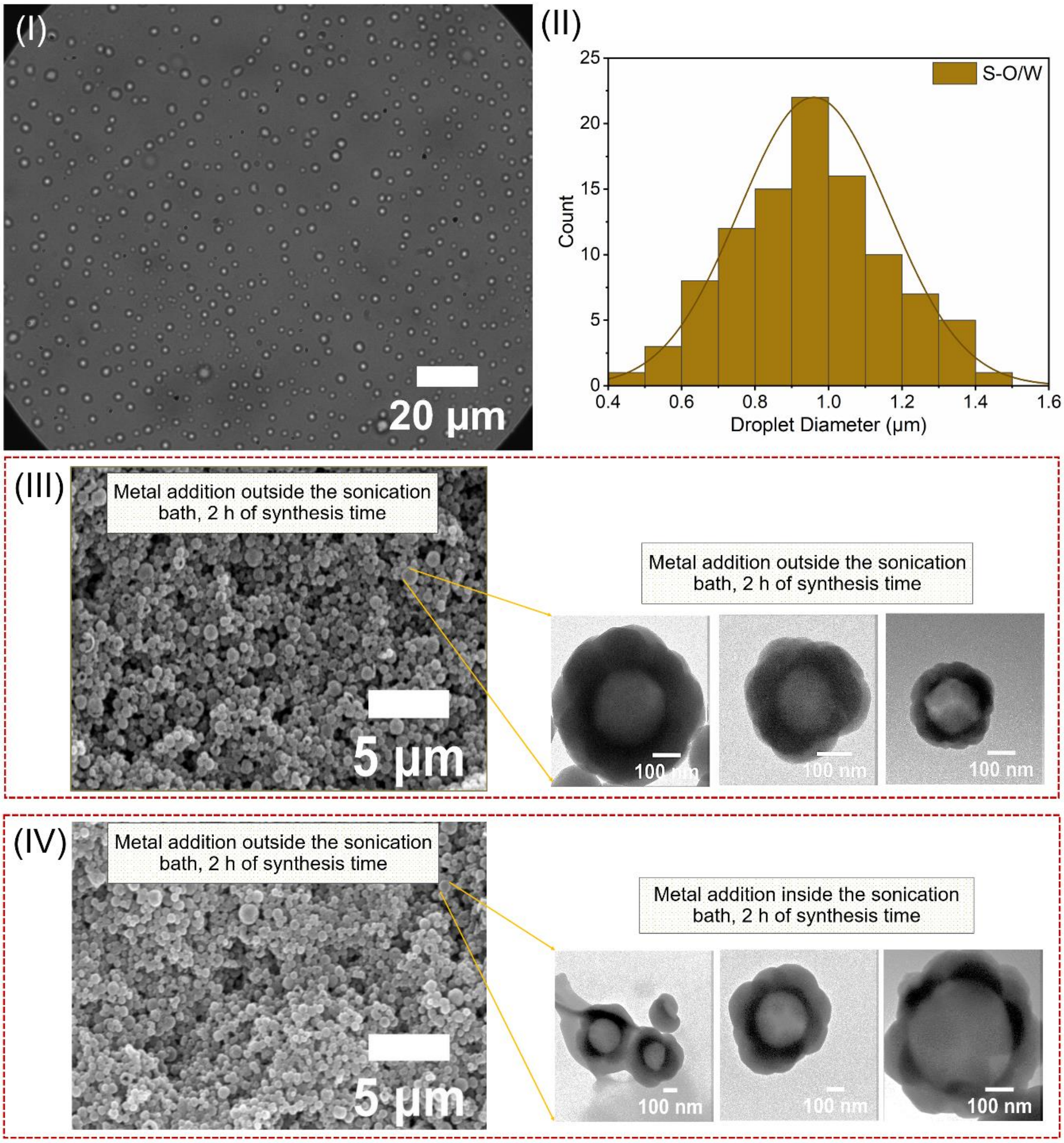

Figure S3. **(I)** Showing the microscopic image of S-O/W emulsion (prepared with 9 v/v% of *n*-hexane and 0.3 g HFS in Mixture-I). **(II)** Droplet size distribution for S-O/W emulsion, **(III)–(IV)** shows the SEM and TEM images for samples ZIF-8-Out and ZIF-8-Ins, respectively.

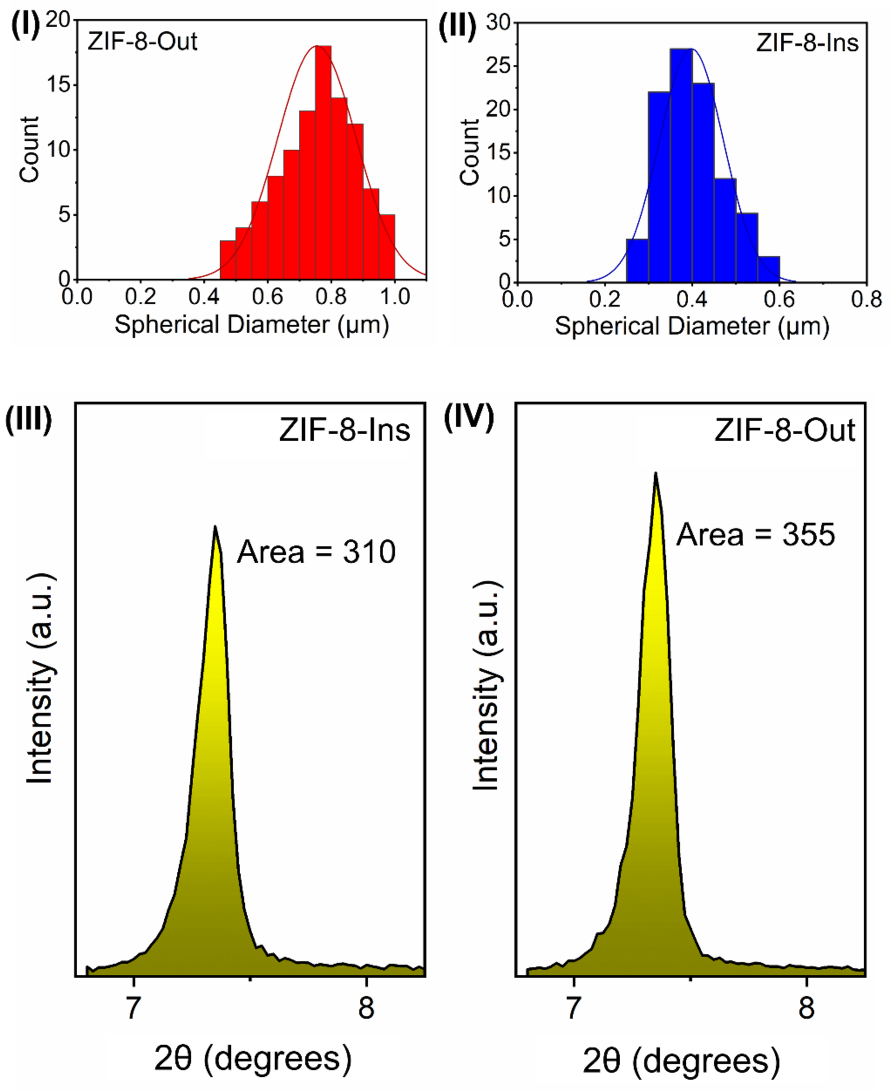

Figure S4. Particle size distribution for **(I)** ZIF-8-Out and **(II)** ZIF-8-Ins. Area under the most prominent XRD peak, i.e., (011), evaluated for **(III)** ZIF-8-Ins and **(IV)** ZIF-8-Out for crystallinity comparison.

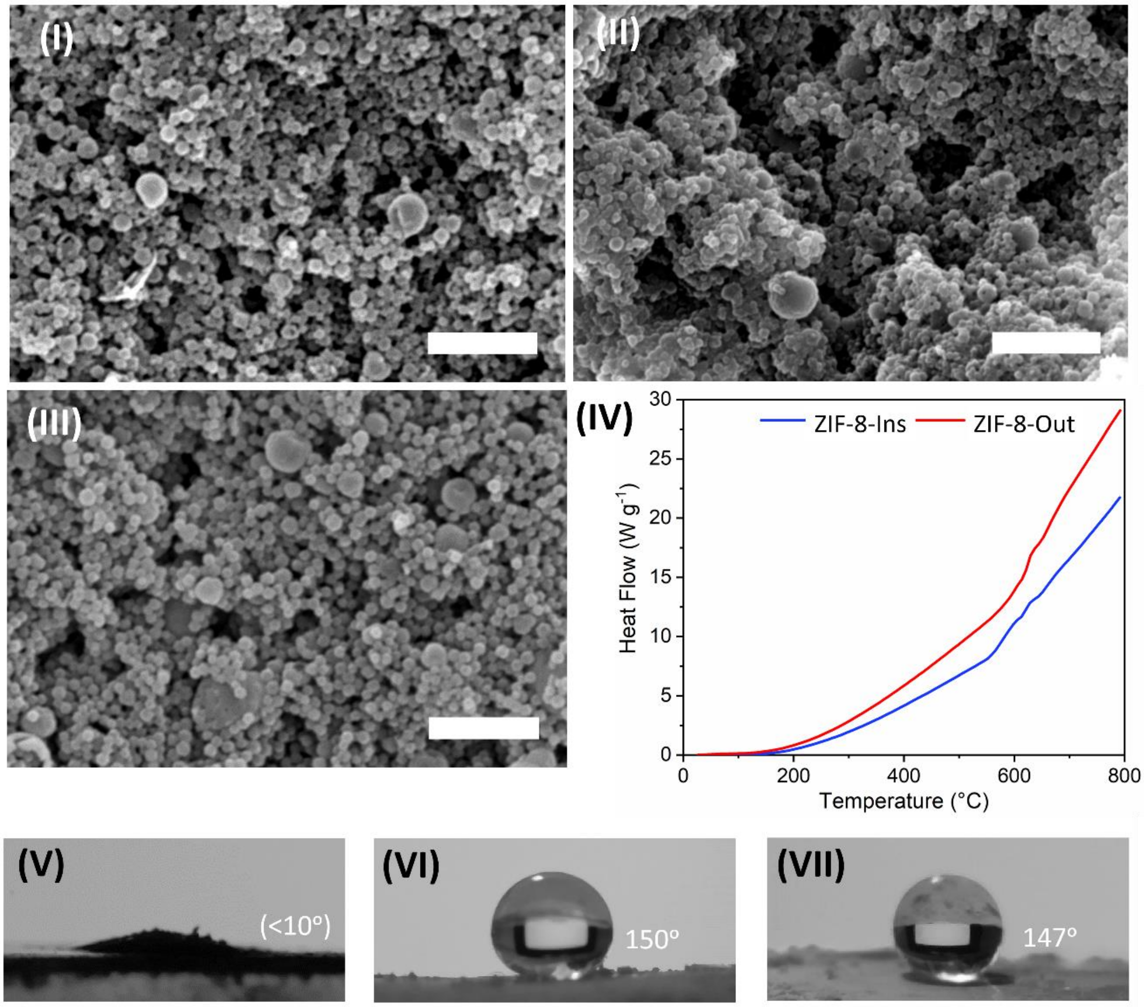

Figure S5. SEM images for samples prepared with **(I)** 13 v/v%, **(II)** 17 v/v%, and **(III)** 23 v/v% of *n*-hexane, respectively, keeping the surfactant (HFS) amount (0.3 g) and the volume of ligand and metal solution (20 mL each) constant. **(IV)** TGA-based DSC pattern for ZIF-8-Ins and ZIF-8-Out. The water contact angle (WCA) for (V) ZIF-8_{Lit}, (VI) Hollow ZIF-8, and (VII) Tube furnaced hollow ZIF-8. (Scale bars are 5 μm)

The TGA-based DSC pattern (Figure 5(IV)) showed similar results (like TGA) with ZIF-8-Ins presenting a higher weight loss, for the same amount of heat influx (W g^{-1}), in comparison to ZIF-8-Out. For instance, at 400 °C, ZIF-8-Ins showed ~13–14% of weight loss for a heat influx of 5 W g^{-1} whereas ZIF-8-Out displayed only ~5% of weight loss for a heat influx of 7 W g^{-1}. The higher weight loss at a lower heat influx value for ZIF-8-Ins suggested the presence of a relatively higher amount of loosely bonded co-solvent, HFS, and/or ligand molecules (as

explained in *Section 3.2.2. Surfactant (HFS) stabilized oil-in-water emulsion (S-O/W) as hollow ZIF-8 growth template: Effect of the amount of oil content*)'.

The water contact angle of the hollow ZIF-8 spheres was studied with the help of Ossila contact angle analyzer (equipment and software) and compared with the ZIF-8_{Lit}. Figure S5 (V)–(VII) presents the water contact angle (WCA) formed by ZIF-8_{Lit}, Hollow ZIF-8, and Tube furnaced Hollow ZIF-8 spheres, respectively. Herein, the samples for WCA were prepared by carefully pressing the as-synthesized powder between the two glass slides for getting a smooth surface of the powder, followed by the addition of a water droplet with the help of a syringe (installed with the apparatus). It was noticed that the as-obtained hollow ZIF-8 spheres showed superhydrophobicity, with a water contact angle (WCA) of 150°, owing to the presence of HFS (as explained in ***Section 3.2.2. Surfactant (HFS) stabilized oil-in-water emulsion (S-O/W) as hollow ZIF-8 growth template: Effect of the amount of oil content***). Comparatively, ZIF-8_{Lit} displayed a super hydrophilic nature with an immediate absorption of the added water droplet and a WCA value as low as <10° (Figure S5 (V)). Later, the hollow ZIF-8 spheres were heated in a tube furnace at 250 °C at a heating rate of 10° min^{-1} in a continuous flow of N_2 gas for 2 hours. The tube furnace heated sample exhibited no significant loss in WCA (147°), highlighting the structural compactness and integrity of the as-prepared hollow ZIF-8 crystalline structure.

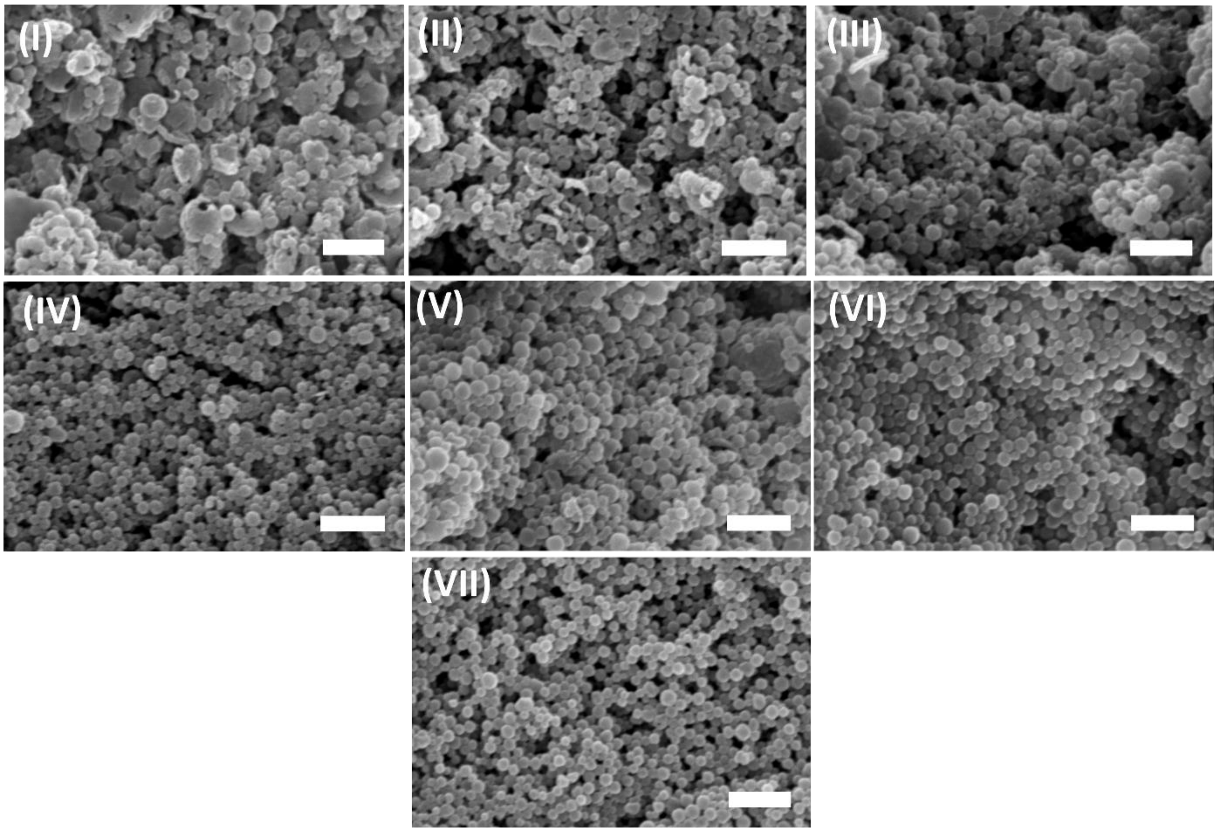

Figure S6. SEM-based synthesis time analysis for **(I)** 0.5 h, **(II)** 1 h, **(III)** 1.5 h, **(IV)** 2 h, **(V)** 6 h, **(VI)**14 h, and **(VII)** 24 h, respectively. (Scale bars are 2 µm)

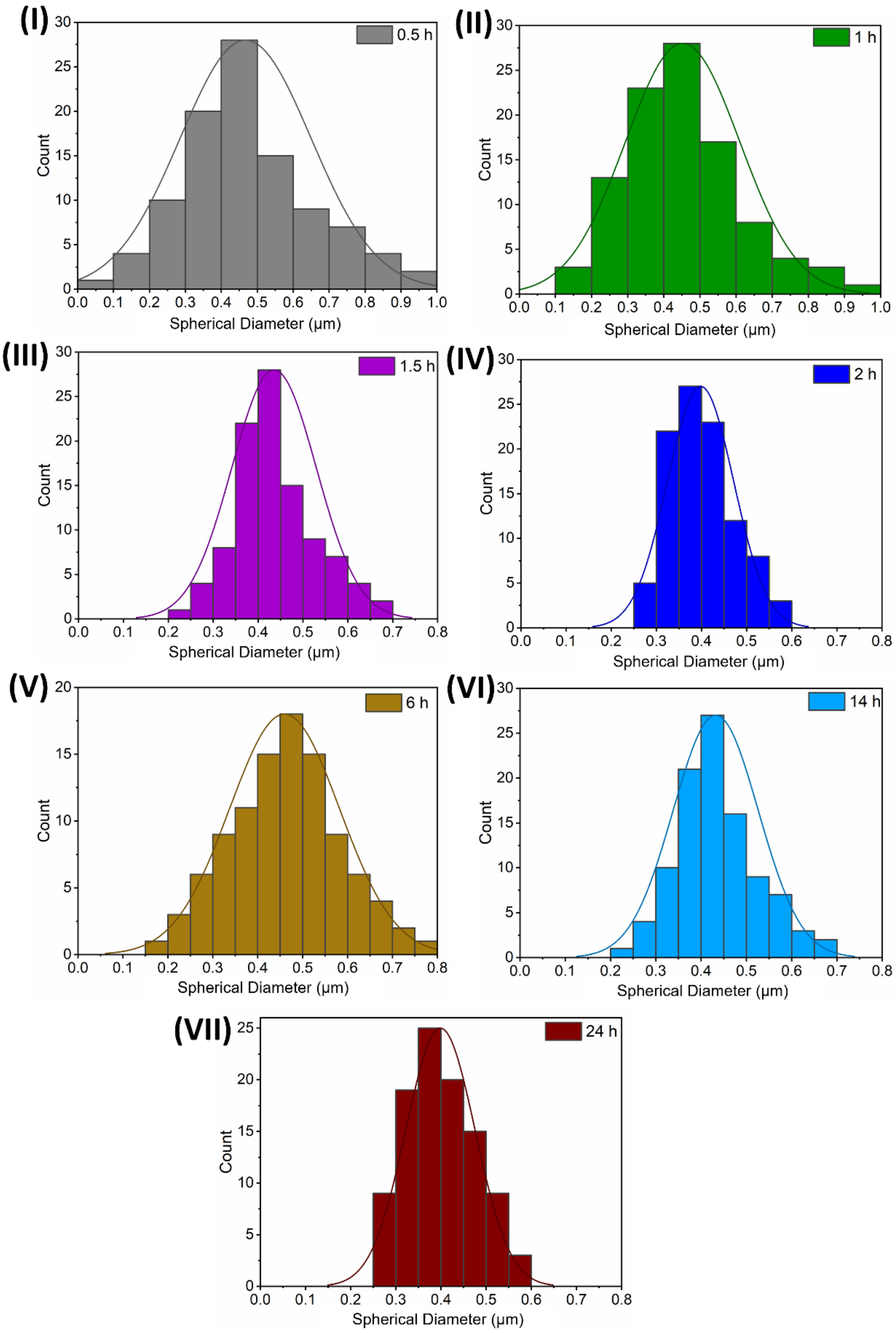

Figure S7. SEM-based sphere-size-distribution for different synthesis times, including **(I)** 0.5 h, **(II)** 1 h, **(III)** 1.5 h, **(IV)** 2 h, **(V)** 6 h, **(VI)** 14 h, and **(VII)** 24 h, respectively.

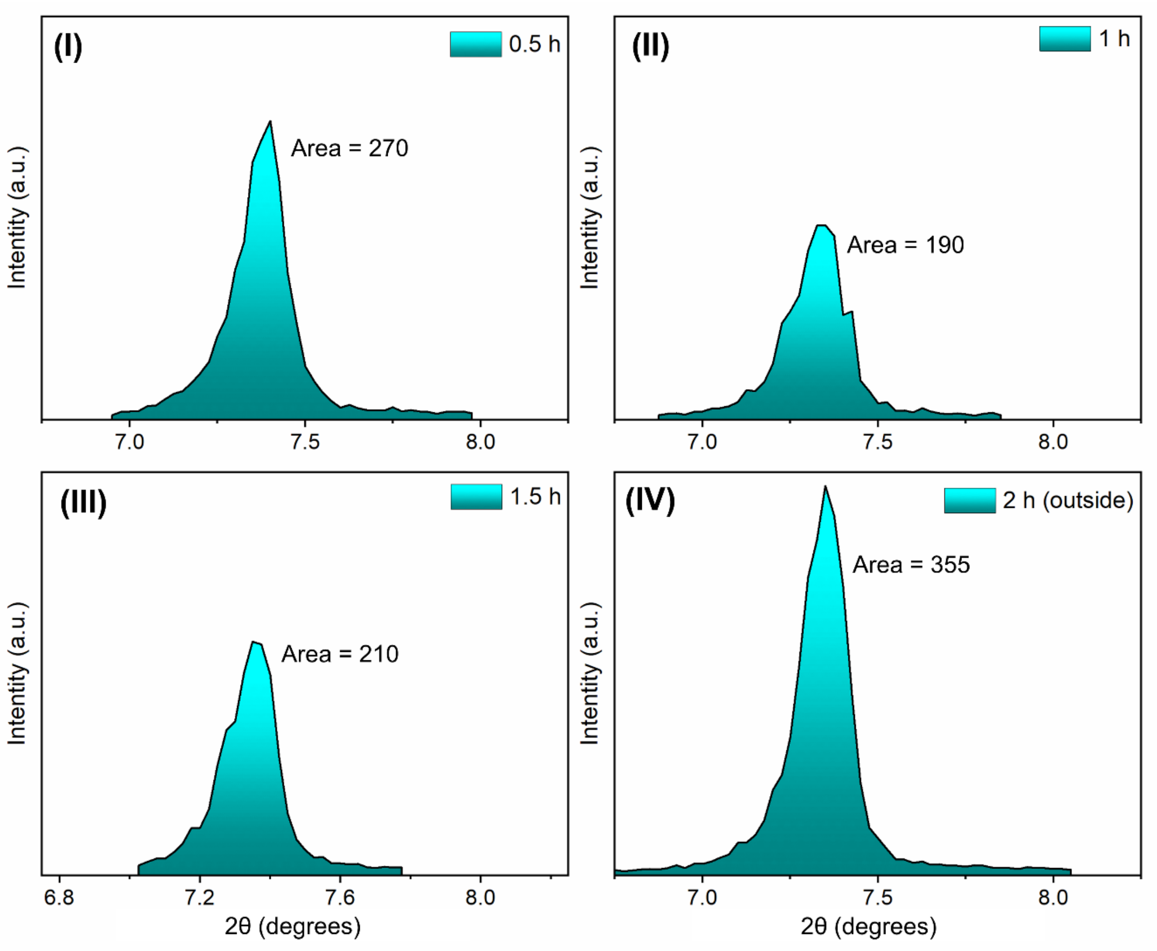

Figure S8. XRD-based synthesis time analysis for **(I)** 0.5 h, **(II)** 1 h, **(III)** 1.5 h, and **(IV)** 2 h (ZIF-8-Out), respectively.

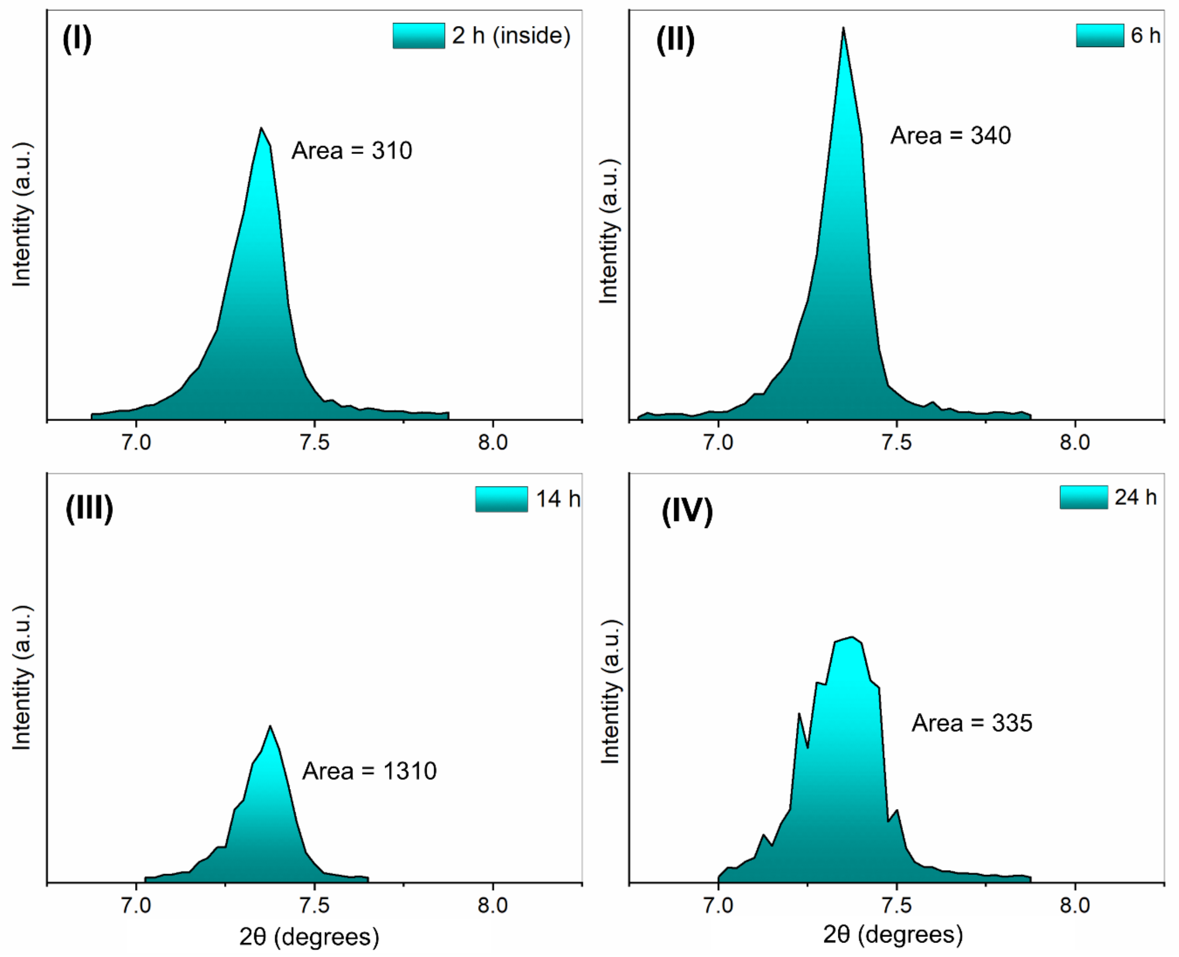

Figure S9. XRD-based synthesis time analysis for **(I)** 2 h (ZIF-8-Ins), **(II)** 6 h, **(III)** 14 h, and **(IV)** 24 h, respectively.

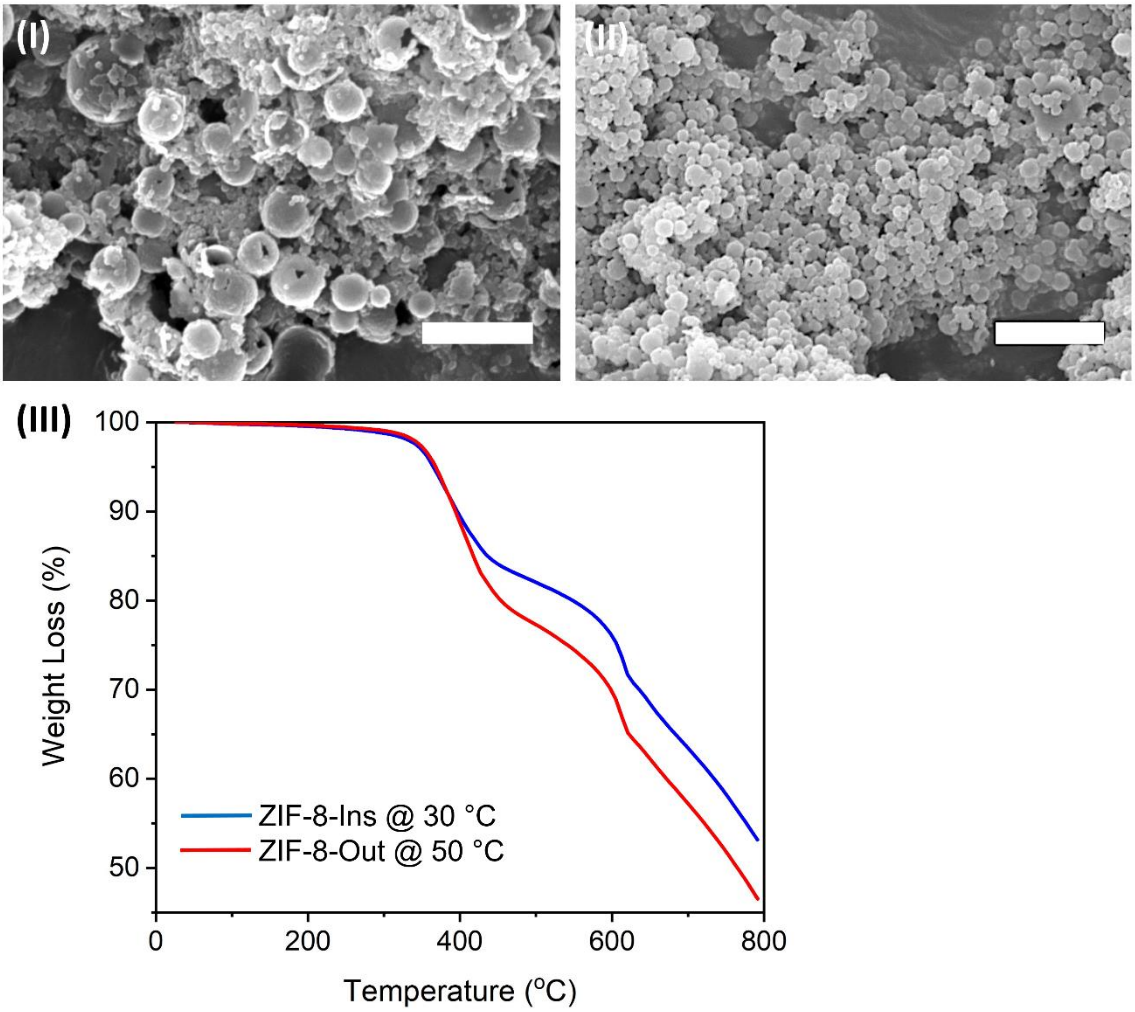

Figure S10. SEM images for high temperature (50 °C) soft-template hollow ZIF-8 synthesis **(I)** in the presence of just *n*-hexane (9 v/v%) without HFS, **(II)** in the presence of both *n*-hexane and HFS, while keeping rest of the synthesis conditions constant. **(III)** TGA comparison between ZIF-8-Out (50 °C) and ZIF-8-Ins (30 °C). (Scale bars are 5 μm)

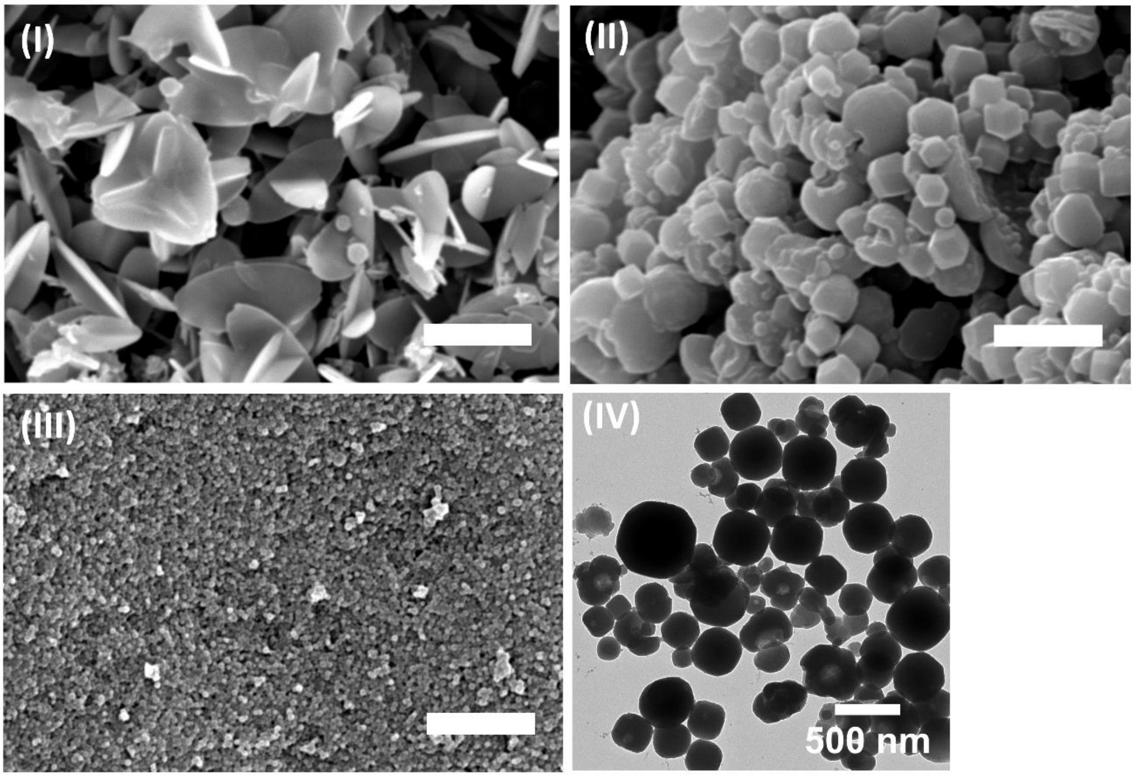

Figure S11. The SEM image showing the case of **(I)** adding the metal solution to the S-O/W mixture at 30 °C, **(II)** doubling the amount of surfactant at 50 °C, and **(III)** the granular morphology for the hollow ZIF-8 sample prepared after doubling the ligand concentration. **(IV)** TEM image for the hollow ZIF-8 sample prepared after doubling the ligand concentration. (Scale bars are 5 μm)

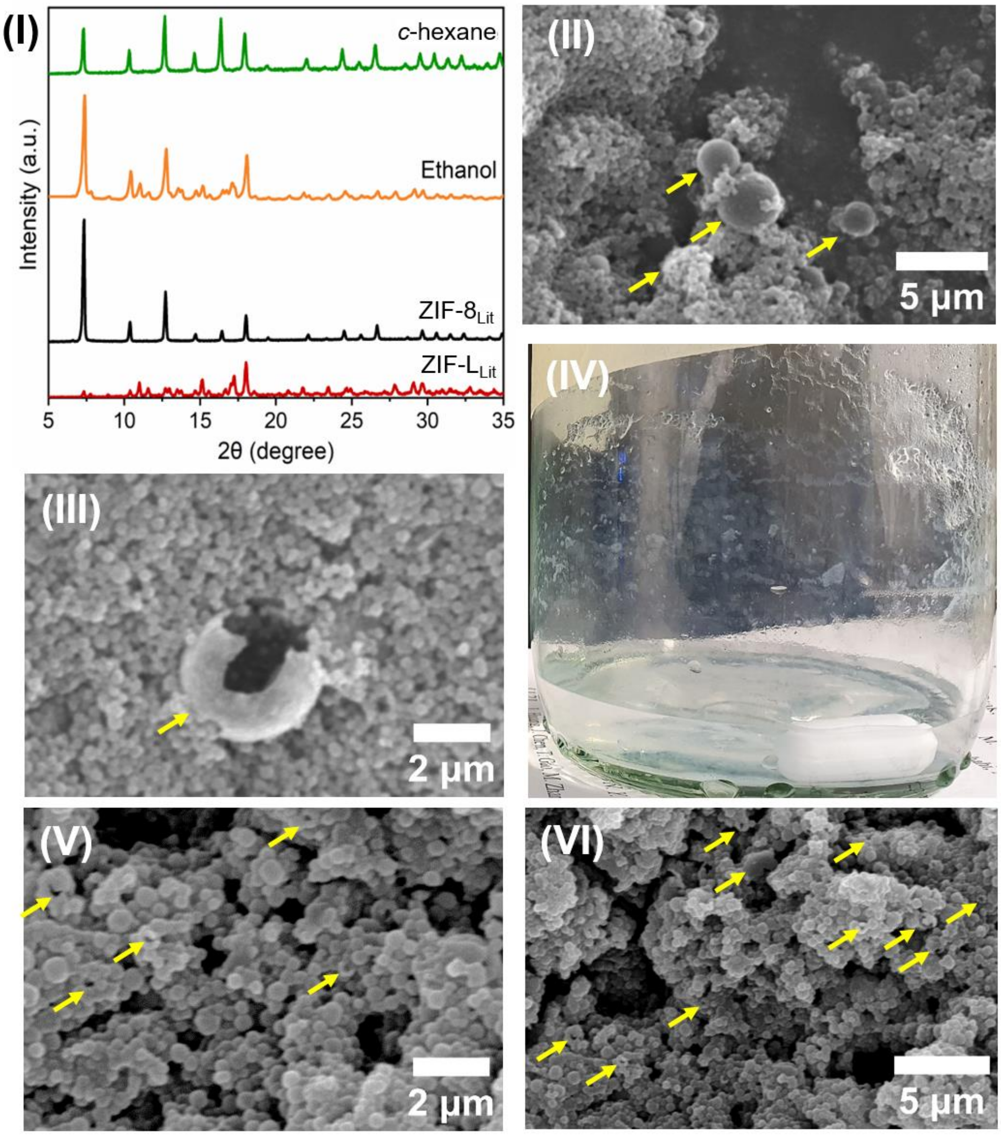

Figure S12. **(I)** The XRD pattern for ZIF-8_{Lit}, ZIF-L_{Lit}, and the sample prepared with ethanol and *c*-hexane. **(II)–(III)** Different magnification SEM images for the samples prepared with ethanol. **(IV)** Amorphous-phase formation upon the use of *n*-dodecane as a co-solvent. **(V)–(VI)** Different magnification SEM images for the samples prepared with *c*-hexane (with yellow arrows pointing toward the hollow spheres and/or broken shells).

Under similar hollow ZIF-8 synthesis conditions, ethanol (9 v/v%) as a co-solvent resulted in the formation of ZIF-L/ZIF-8 mixed-phase, as shwn from the XRD pattern in Figure S12 (I). Despite showing the ZIF-8 characteristic XRD peaks, the presence of the ZIF-L character

shows incomplete phase conversion with the available amount of ethanol. In our previously reported work, a minimum of 25 v/v% of ethanol was required for a pure ZIF-8 phase formation.[4] Therefore, more amount of ethanol will be needed for ZIF-8 phase formation in comparison to *n*-hexane which requires only 9 v/v% of overall concentration, resulting in lower solvent consumption. Moreover, the SEM images provided in Figure S12 (II)–(III) present the formation of a mixed morphology, containing both solid and hollow spheres. The above-mentioned XRD and SEM images suggested that a higher concentration (v/v%) of ethanol will be needed as a co-solvent for the possible hollow sphere formations having a single ZIF-8 phase. In contrast, the addition of 9 v/v% (4 mL) of *n*-dodecane resulted in the formation of a jelly-like substance with no solid crystal formation,[5] suggesting the presence of an amorphous phase, as shown in Figure S12 (IV). This phenomenon can be attributed to the relatively higher viscosity of *n*-dodecane, which prohibited a facile diffusion of reagent molecules,[6] resulting in the formation of a thick phase. Possibly, a relatively lower concentration of *n*-dodecane will prove effective for the desired ZIF-8 synthesis.

Interestingly, unlike ethanol and *n*-dodecane, the use of *c*-hexane as a co-solvent resulted in the formation of a pure ZIF-8 phase, as shown by the XRD pattern in Figure S12 (I). Moreover, the SEM images presented the formation of a mixed morphology with both solid and hollow sphere formations (Figure S12 (V)–(VI)). A relatively higher concentration of *c*-hexane, or the variation in the amount of HFS, as a surfactant, will be required for promoting the hollow sphere formations. Hence, the current synthesis can be regarded as an optimized recipe when only *n*-hexane is used as a co-solvent. This further highlights the existence of a relatively stronger *n*-hexane-to-ligand interaction in maintaining the microemulsion template for ZIF-8 growth in comparison to the other co-solvents.

Table S1. The effect of variation in the oil (*n*-hexane) volume on hollow ZIF-8 synthesis.

**Oil Volume (mL)**	**Oil Volume Per Cent (v/v%)**	**Morphology**	**Dimensionality (D)**	**Hollow Sphere Average Diameter (µm)**	**Standard Deviation**
0	0	Leaf-shaped	2	–	–
0.5	1	Solid	3	–	–
1	3	Solid	3	–	–
2	5	Solid	3	–	–
4	9	Solid, Hollow	0, 3	2.50	0.95
8	17	Solid, Hollow	0, 3	1.90	0.55
12	23	Solid, Hollow	0, 3	0.90	0.20

Table S2. The effect of variation in the oil (*n*-hexane) volume (keeping the surfactant amount constant at 0.3 g) on Hollow ZIF-8 synthesis.

Oil Volume (mL)	Oil Volume Per Cent (v/v%)	Surfactant/Oil (S/O) Ratio (g mL^{-1})	Morphology	Hollow Sphere Average Diameter (μm)	Standard Deviation
0	0	–	Leaf-shaped	–	–
1	3	0.30	Solid, Hollow	1.00	0.30
1.5	4	0.20	Solid, Hollow	1.00	0.25
2	5	0.15	Mostly hollow	1.20	0.35
4 (ZIF-8-Out)	9	0.075	Hollow	0.75	0.14
4 (ZIF-8-Ins)	9	0.075	Hollow	0.40	0.08
6	13	0.05	Mostly hollow	0.90	0.28
8	17	0.037	Mostly hollow	0.96	0.40
12	23	0.025	Mostly hollow	1.40	0.45

Table S3. The effect of variation in the amount of surfactant, keeping the *n*-hexane concentration constant (9 v/v%), on Hollow ZIF-8 fabrication.

Amount of Surfactant (g)	Surfactant/Oil (S/O) Ratio (g mL^{-1})	Morphology	Hollow Sphere Average Diameter (μm)	Standard Deviation
0	0	Solid, Hollow	2.50	0.95
0.10	0.025	Mostly hollow	1.50	0.55
0.15	0.037	Mostly hollow	1.45	0.60
0.25	0.063	Mostly hollow	1.30	0.35
0.30 (ZIF-8-Out)	0.075	Hollow	0.75	0.14
0.30 (ZIF-8-Ins)	0.075	Hollow	0.40	0.08
0.40	0.10	Solid, Hollow	0.37	0.10
0.60	0.15	Mostly solid	0.25	0.10

Table S4. The effect of synthesis time on soft template hollow ZIF-8 nanosphere average diameter for a constant surfactant/oil ratio (0.075 g mL^{-1}).

Synthesis Time (h)	Surfactant/Oil Ratio (g mL^{-1})	Morphology	Hollow Sphere Average Diameter (µm)	Standard Deviation
0.5	0.075	Hollow	0.50	0.30
1		Hollow	0.45	0.20
1.5		Hollow	0.43	0.15
2		Hollow	0.40	0.08
6		Solid, Hollow	0.47	0.12
14		Solid, Hollow	0.45	0.10
24		Mostly solid	0.40	0.08

Table S5. The effect of synthesis time time on material's crystallinity, estimated by area under the most prominent peak (011) of the XRD curve. The analysis was performed for the duration of 0.5 h, 1 h, 1.5 h, 2 h, 6 h, 14 h, and 24 h of synthesis time and a constant surfactant/oil ratio (0.075 g mL^{-1}).

Synthesis Time (h)	Surfactant/Oil Ratio (g mL^{-1})	Area Under the Curve
0.5	0.075	270
1		190
1.5		210
2		310
6		340
14		130
24		335

Table S6. The effect of high-temperature synthesis on synthesized hollow ZIF-8 nanosphere's morphology prepared with 9 v/v% (4 mL) of *n*-hexane.

**Surfactant/Oil Ratio (g mL^{-1})**	**Metal addition**	**Synthesis Temperature (°C)**	**Morphology**	**Hollow Sphere Average Diameter (μm)**	**Standard Deviation**
0	Outside	30	Solid, Hollow	2.50	0.95
0	Outside	50	Solid, Hollow	1.80	0.30
0.075	Outside	30	Hollow	0.75	0.14
0.075	Outside	50	Hollow	0.65	0.10
0.075	Inside	30	Inside	0.40	0.08

Table S7. ZIF-8 gas (CO_2) adsorption comparison with the previous literature

Literature reference	Sample	Surface area ($m^2\ g^{-1}$)	Total pore volume ($cm^3\ g^{-1}$)	CO_2 adsorption capacity
Z. Zhang et al.[7]	ZIF-8	1025	0.45	225 mg g^{-1} (298 K, 30 bar)
S. Xian et al.[8]	ZIF-8	1150	–	0.75 mmol g^{-1} (298 K, P/P_o = 1)
J. Pokhrel et al.[9]	ZIF-8	1318	–	0.65 mmol g^{-1} (303 K, 900 mbar)
Z. Zhang et al.[10]	ZIF-8	1025	0.54	225 mg g^{-1} (298 K, 25 bar)
N. H. b. Yahya et al.[11]	ZIF-8	–	–	0.6673 mmol g^{-1} (298 K, 1 bar)
J. McEwen et al.[12]	ZIF-8	1475	0.70	0.75 mmol g^{-1} (298 K, 900 mbar)
Current work	ZIF-8-Out (Outside, 2 h)	1110	0.64	2.24 mmol g^{-1} (273 K, 1.75 bar)
Current work	ZIF-8-Out (Outside, 2 h)	1110	0.64	1.14 mmol g^{-1} (298 K, 1.75 bar)
Current work	ZIF-8-Ins (Inside, 2 h)	1325	0.78	2.19 mmol g^{-1} (273 K, 1.75 bar)
Current work	ZIF-8-Ins (Inside, 2 h)	1325	0.78	1.07 mmol g^{-1} (298 K, 1.75 bar)
Current work	ZIF-8 (outside, 6 h)	1470	0.95	–

Table S8. Comparison of hard-/soft-templating synthesis of hollow ZIF-8

Author	Hard/soft template	Sample	Fabrication methodology	Synthesis time (h)	Synthesis temperature (°C)	BET (m^2 g^{-1})	Total pore volume (cm^3 g^s)	Application
M. Zhang et al.[13]	Hard	TiO_2@ZIF-8	Double-shell growth via sono-crystallization	>24	500	1123	0.62	Hydrogen generation
S. Hwang et al.[14]	Hard	ZIF-8	Solvothermal surface coating followed by selective core removal	>24	80	1376.2-1528.5	–	Gas separation
J. H. Lee et al.[15]	Hard	ZIF-8	Solvothermal synthesis followed by self-sacrifice/excavation	>24	90	1502	0.89	Gas separation
H. Liu et al.[16]	Hard	HRP@H-ZIF-8-GOx	Immobilizing HRP and GOx in hollow ZIF-8 cavity	6	25	–	–	Colourimetric detections
Y. Zhao et al.[17]	Hard	Pd@ZIF-8	PVP as a nucleation substrate for ZIF-8 growth	4	RT	927	–	Catalytic properties
J. Yang et al.[18]	Hard	ZIF-67@ZIF-8	Phase transformation in an organic solvent	4	40	1027	–	Acetylene semi-hydrogenation
K.-k. Sun et al.[19]	Hard	Co@NC(ZIF-8)	Solvothermal synthesis followed by sacrificial growth	12	35	1042	1.25	synthesis of nitriles
H. J. Lee et al.[20]	Hard	ZIF-8	Core-shell growth with subsequent core removal	0.15	70	–	–	–
X. Cheng et al.[21]	Soft	ZIF-8	Interfacial synthesis method supported by oil in water emulsion	>24	RT	1408	–	Water-selective permeation
Y. Yang et al.[22]	Soft	ZIF-8	Nanoemulsion	>24	RT	1098	–	Catalysis
This Work	Soft	ZIF-8 (Outside,6h)	Oil in water emulsion supported by surfactant and sonication	2	30	1470	0.95	Gas separation
This Work	Soft	ZIF-8 (Inside,2h)	Oil in water emulsion supported by surfactant and sonication	2	30	1325	0.78	Gas separation
This Work	Soft	ZIF-8 (Outside,2h)	Oil in water emulsion supported by surfactant and sonication	2	30	1110	0.64	Gas separation

REFERENCES

(1) Chen, R.; Yao, J.; Gu, Q.; Smeets, S.; Baerlocher, C.; Gu, H.; Zhu, D.; Morris, W.; Yaghi, O. M.; Wang, H. A Two-Dimensional Zeolitic Imidazolate Framework with a Cushion-Shaped Cavity for CO_2 Adsorption. *Chem. Commun.* **2013**, *49*, 9500–9502.

(2) Pan, Y.; Liu, Y.; Zeng, G.; Zhao, L.; Lai, Z. Rapid Synthesis of Zeolitic Imidazolate Framework-8 (ZIF-8) Nanocrystals in an Aqueous System. *Chem. Commun.* **2011**, *47*, 2071–2073.

(3) Salihovic, M.; Zickler, G. A.; Fritz-popovski, G.; Ulbricht, M.; Paris, O.; Hüsing, N.; Presser, V.; Elsaesser, M. S. Reversibly Compressible and Freestanding Monolithic Carbon Spherogels. *Carbon* **2019**, *153,* 189–195.

(4) Lewis, A.; Butt, F. S.; Wei, X.; Mazlan, N. A.; Chen, Z.; Yang, Y.; Yang, S.; Radacsi, N.; Chen, X.; Huang, Y. Crystallization and phase selection of zeolitic imidazolate frameworks in aqueous cosolvent systems: The role and impacts of organic solvents. *Results Eng.* **2023**, *17*, 100751.

(5) Lv, X.; Sullivan, P.; Feng, D. MOFs the Movie: Molecule to Nuclei Evolution during Metal-Organic Framework Formation. *Matter* **2022**, *5*, 11–13.

(6) Song, K.; Koo, J. Y.; Choi, H. C. Viscosity Effect on the Strategic Kinetic Overgrowth of Molecular Crystals in Various Morphologies: Concave and Octapod Fullerene Crystals. *RSC Adv.* **2021**, *11*, 20992-20996.

(7) Zhang, Z.; Xian, S.; Xi, H.; Wang, H.; Li, Z. Improvement of CO_2 Adsorption on ZIF-8 Crystals Modified by Enhancing Basicity of Surface. *Chem. Eng. Sci.* **2011**, *66*, 4878–4888.

(8) Xian, S.; Xu, F.; Ma, C.; Wu, Y.; Xia, Q.; Wang, H.; Li, Z. Vapor-Enhanced CO_2 Adsorption Mechanism of Composite PEI@ZIF-8 Modified by Polyethyleneimine for CO_2/N_2 Separation. *Chem. Eng. J.* **2015**, *280*, 363–369.

(9) Pokhrel, J.; Bhoria, N.; Anastasiou, S.; Tsoufis, T.; Gournis, D.; Romanos, G.; Karanikolos, G. N. CO_2 Adsorption Behavior of Amine-Functionalized ZIF-8, Graphene Oxide, and ZIF-8/Graphene Oxide Composites under Dry and Wet Conditions. *Microporous Mesoporous Mater.* **2018**, *267*, 53–67.

(10) Qiao, Z.; Wang, Z.; Zhang, C.; Yuan, S.; Zhu, Y.; Wang, J.; Wang, S. PVAm–PIP/PS Composite Membrane with High Performance for CO_2/N_2 Separation. *AIChE J.* **2012**, *59*, 215–228.

(11) Yahya, N. H. B.; Yeong, Y. F.; Lai, L. S. Synthesis of Amino-Impregnated ZIF-8 for CO_2 Adsorption. *IOP Conf. Ser. Mater. Sci. Eng.* **2017**, *226*, 012164.

(12) McEwen, J.; Hayman, J. D.; Ozgur Yazaydin, A. A Comparative Study of CO_2, CH4 and N_2 Adsorption in ZIF-8, Zeolite-13X and BPL Activated Carbon. *Chem. Phys.* **2013**, *412*, 72–76.

(13) Zhang, M.; Shang, Q.; Wan, Y.; Cheng, Q.; Liao, G.; Pan, Z. Self-Template Synthesis of Double-Shell TiO_2@ZIF-8 Hollow Nanospheres via Sonocrystallization with Enhanced Photocatalytic Activities in Hydrogen Generation. *Appl. Catal. B Environ.* **2019**, *241*, 149–158.

(14) Hwang, S.; Chi, W. S.; Lee, S. J.; Im, S. H.; Kim, J. H.; Kim, J. Hollow ZIF-8 Nanoparticles Improve the Permeability of Mixed Matrix Membranes for CO_2/CH_4 Gas Separation. *J. Memb. Sci.* **2015**, *480*, 11–19.

(15) Lee, J. H.; Kwon, H. T.; Bae, S.; Kim, J.; Kim, J. H. Mixed-Matrix Membranes Containing Nanocage-like Hollow ZIF-8 Polyhedral Nanocrystals in Graft Copolymers for Carbon Dioxide/Methane Separation. *Sep. Purif. Technol.* **2018**, *207*, 427–434.

(16) Liu, H.; Du, Y.; Gao, J.; Zhou, L.; He, Y.; Ma, L.; Liu, G.; Huang, Z.; Jiang, Y. Compartmentalization of Biocatalysts by Immobilizing Bienzyme in Hollow ZIF-8 for Colorimetric Detection of Glucose and Phenol. Ind. Eng. *Chem. Res.* **2020**, *59*, 42–51.

(17) Zhao, Y.; Ni, X.; Ye, S.; Gu, Z. G.; Li, Y.; Ngai, T. A Smart Route for Encapsulating Pd Nanoparticles into a ZIF-8 Hollow Microsphere and Their Superior Catalytic Properties. *Langmuir* **2020**, *36*, 2037–2043.

(18) Yang, J.; Zhang, F.; Lu, H.; Hong, X.; Jiang, H.; Wu, Y.; Li, Y. Hollow Zn/Co ZIF Particles Derived from Core-Shell ZIF-67@ZIF-8 as Selective Catalyst for the Semi-Hydrogenation of Acetylene. *Angew. Chemie* **2015**, *127*, 11039–11043.

(19) Sun, K. K.; Sun, J. L.; Lu, G. P.; Cai, C. Enhanced Catalytic Activity of Cobalt Nanoparticles Encapsulated with an N-Doped Porous Carbon Shell Derived from Hollow ZIF-8 for Efficient Synthesis of Nitriles from Primary Alcohols in Water. *Green Chem.* **2019**, *21*, 4334–4340.

(20) Lee, H. J.; Cho, W.; Oh, M. Advanced Fabrication of Metal-Organic Frameworks: Template-Directed Formation of Polystyrene@ZIF-8 Core-Shell and Hollow ZIF-8 Microspheres. *Chem. Commun.* **2012**, *48*, 221–223.

(21) Cheng, X.; Jiang, Z.; Cheng, X.; Yang, H.; Tang, L.; Liu, G.; Wang, M.; Wu, H.; Pan, F.; Cao, X. Water-Selective Permeation in Hybrid Membrane Incorporating Multi-Functional Hollow ZIF-8 Nanospheres. *J. Memb. Sci.* **2018**, *555*, 146–156.

(22) Yang, Y.; Wang, F.; Yang, Q.; Hu, Y.; Yan, H.; Chen, Y. Z.; Liu, H.; Zhang, G.; Lu, J.; Jiang, H. L.; Xu, H. Hollow Metal-Organic Framework Nanospheres via Emulsion-Based Interfacial Synthesis and Their Application in Size-Selective Catalysis. *ACS Appl. Mater. Interfaces* **2014**, *6*, 18163–18171.
